# Analysis of high-identity segmental duplications in the grapevine genome

**DOI:** 10.1186/1471-2164-12-436

**Published:** 2011-08-26

**Authors:** Giuliana Giannuzzi, Pietro D'Addabbo, Marica Gasparro, Maurizio Martinelli, Francesco N Carelli, Donato Antonacci, Mario Ventura

**Affiliations:** 1 Department of Biology, University of Bari, Bari 70126, Italy; 2 Agricultural Research Council, Research Unit for Table Grapes and Wine Growing in Mediterranean Environment (CRA-UTV), Turi (BA) 70010, Italy; 3 National Institute of Nuclear Physics (INFN), Bari 70126, Italy; 4 Department of Physics, University of Bari, Bari 70126, Italy; 5 Department of Genome Sciences, University of Washington School of Medicine, Seattle, Washington 98195, USA

## Abstract

**Background:**

Segmental duplications (SDs) are blocks of genomic sequence of 1-200 kb that map to different loci in a genome and share a sequence identity > 90%. SDs show at the sequence level the same characteristics as other regions of the human genome: they contain both high-copy repeats and gene sequences. SDs play an important role in genome plasticity by creating new genes and modeling genome structure. Although data is plentiful for mammals, not much was known about the representation of SDs in plant genomes. In this regard, we performed a genome-wide analysis of high-identity SDs on the sequenced grapevine (*Vitis vinifera*) genome (PN40024).

**Results:**

We demonstrate that recent SDs (> 94% identity and >= 10 kb in size) are a relevant component of the grapevine genome (85 Mb, 17% of the genome sequence). We detected mitochondrial and plastid DNA and genes (10% of gene annotation) in segmentally duplicated regions of the nuclear genome. In particular, the nine highest copy number genes have a copy in either or both organelle genomes. Further we showed that several duplicated genes take part in the biosynthesis of compounds involved in plant response to environmental stress.

**Conclusions:**

These data show the great influence of SDs and organelle DNA transfers in modeling the *Vitis vinifera *nuclear DNA structure as well as the impact of SDs in contributing to the adaptive capacity of grapevine and the nutritional content of grape products through genome variation. This study represents a step forward in the full characterization of duplicated genes important for grapevine cultural needs and human health.

## Background

Grapevine (*Vitis vinifera*) is one of the oldest (appeared approximately 65 million years ago) and most important fruit crops in the world [1]. Today, this species is widely cultivated and represents almost the 98% of grape vineyards subdivided into table, wine and raisin grapes [2]. The productivity is generally valuated only by phenotype observation, although it is largely influenced by genotype, environment and cultural techniques. Grape was shown to reduce the incidence of cardiovascular and other diseases due to the content of secondary metabolites such as resveratrol, quercetin and others polyphenols [3].

The grapevine genome is diploid and organized in 38 chromosomes (n = 19), with a total size of ~487 Mb. A genotype originally derived from the Pinot Noir grape variety (PN40024) has recently been sequenced and assembled using a whole-genome shotgun (WGS) approach resulting in 12-fold coverage [4].

It was reported that during plant and animal genome evolution, whole-genome and segmental duplication (SD) events occurred leading to an increase in biological complexity and the origin of evolutionary novelties [5,6]. In fact, gene duplication represents the primary source of new gene function origination [7-11]. SDs are large blocks of genomic sequence at least 1 kb in size mapping to more than one location in a genome. Highly similar SDs are regions of genome instability as they predispose chromosomes to rearrangements providing templates for non-allelic homologous recombination (NAHR) events. The erroneous pairing between two non-allelic SDs leads, after crossover, to translocation, inversion, deletion or duplication [12]. Notably in plants, previous studies have reported a large impact of SDs on the evolution of genes involved in disease resistance, berry development and the ripening process [13-15]. An example is the NBS-LRR gene family, whose evolution and expansion through duplication have been studied in the *Arabidopsis thaliana *genome [14,16].

It is widely known that the identification and characterization of high-identity SDs is problematic in WGS-based sequencing. The inability to identify such duplications results in the merging of distinct duplicated loci into the same sequence. More divergent duplications with < 94% sequence identity can be easily resolved by the WGS assembly method, whereas high-identity duplications (> 94%) are frequently collapsed [17,18]. Studies about the role of SD in *Vitis vinifera *and other plant genome evolution have followed classical assembly-based approaches of sequence alignment and comparison [4,19-22], thus ignoring the impact and contribution of highly similar SDs.

The whole-genome shotgun sequence detection (WSSD) method is a genome-wide approach identifying large (>= 10 kb in length), high-identity (> 94%) SDs based on their higher depth of coverage of WGS sequence reads aligned to the reference genome sequence, in an assembly-independent fashion [23]. This approach was used to evaluate the genomic architecture of recent SDs in human, mouse, chimpanzee, dog and bovine genomes, all species belonging to the mammalian group [18,23-26]. Genome-wide analysis of large, high-identity SDs in plant genomes has never been reported. Therefore, the extent and organization of highly similar SDs in any sequenced plant genome are not known.

In this work, we present an analysis of *Vitis vinifera *PN40024 inbred line genome architecture and its high-identity duplication content. We generated an SD map for this genome and discovered that 85 Mb of grapevine genome were duplicated. In this way, we identified duplicated regions that might have been misassembled or erroneously merged in the current genome assembly. We detected 2,589 genes embedded in the identified duplicated segments, demonstrating a role of duplication in the evolution of these genes. Furthermore, the identified genomic regions are candidate hot spots for *de novo *duplication and/or copy number variation among the wide list of existing grapevine varieties and may underlie the molecular basis of some phenotypical differences among them.

## Results

We applied the WSSD strategy [23] to the PN40024 grapevine genome to detect SDs (>= 10 kb in length, > 94% sequence identity) based on a read depth methodology. Plant genomes are enriched in repetitive elements, which impose problems in SD detection since large high-copy common repeats may be erroneously classified as SDs. To circumvent this issue, we sought to establish the best repeat masking parameters for the grapevine genome. We compared three different settings of the RepeatMasker and Tandem Repeats Finder (TRF) softwares: i) known repeats with < 10% divergence from the consensus sequence and tandem repeats converted to lowercase, as performed in previous WSSD analyses ("div10_low" method) [24-26]; ii) known repeats without a divergence threshold and tandem repeats converted to lowercase ("nodiv_low" method); and iii) known repeats without a divergence threshold and tandem repeats converted to N ("nodiv_N" method). These three methods differ for the stringency of repeat masking (divergence < 10% vs. no divergence threshold) and the possibility of extending the alignment through masked sequence to reach the alignment length threshold (lowercase vs. N masking). 12.28% of the *Vitis *genome was masked with a threshold divergence equal to 10, whereas 29.26% was masked with no divergence threshold. Less stringent masking (i.e. without a divergence threshold) not only reduces the genomic sequence available for read matches, but also increases the effective size of 5 kub (kilo of unmasked bases) windows, which comprise 5 kb of unmasked nucleotides plus the interposed masked ones. In fact, the effective window size depends on the prevalence of masked sequence in the corresponding region.

To experimentally estimate the duplication content in *Vitis vinifera *and establish a control set for WSSD analysis, we randomly selected 100 BAC clones from the Pinot Noir VVPN40024 library to use as probes in FISH (fluorescent *in situ *hybridization) experiments. We examined hybridization signals on both interphase and metaphase chromosomes to evaluate the single or duplicated nature of the corresponding genomic regions. We based estimations on the observation of at least 50 nuclei.

**Figure 1:**
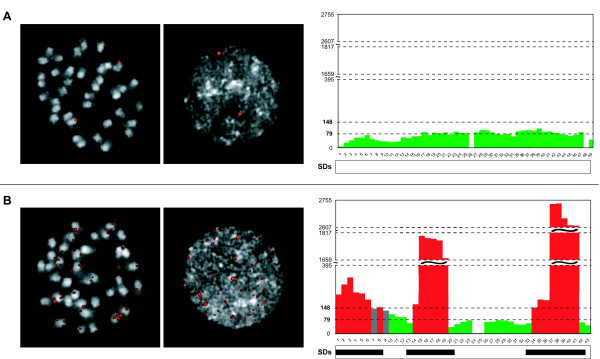
**FISH results and WSSD outputs for a single and a duplicated BAC clone**. FISH signals on metaphase and interphase chromosomes (left panel), and WSSD coverages and segmentally duplicated regions according to the nodiv_N method of the genomic loci (right panel) of VV40024H153C18 single **(A) **and VV40024H153D21 duplicated **(B) **clones.

We distinguished signal patterns in single (one or two), duplicated (more than two), tandem-duplicated (more than two and on the same chromosome), and undefined according to the number and pattern of observed signals (Figure 1, Additional file 1). A pattern was assigned as "undefined" when the copy number could not be estimated because of the high background or the pattern was not consistent among the observed nuclei. The results revealed 45 single, 21 duplicated, 5 tandem-duplicated, and 16 undefined BACs, whereas 13 clones gave no result (Additional file 2). All tandem-duplicated clones showed four clusters in both nuclei and metaphases and mapped to two pairs of chromosomes (Additional file 1).

End sequences from seventy-nine BAC clones were mapped on the grapevine genome assembly, where eight mapped by one end only (one-end anchored, OEA) (Additional file 2). The five tandemly duplicated BAC clones were not anchored to the genome assembly. BAC end sequences (BES) of these clones were highly similar when aligned to the BES of the other tandemly duplicated clones, except 153C07FM1, with an average identity of 93.59% (Additional file 3). Sequence similarities and FISH co-hybridization results revealed that all tandem-duplicated BACs hybridize to the same chromosomal region.

**Figure 2:**
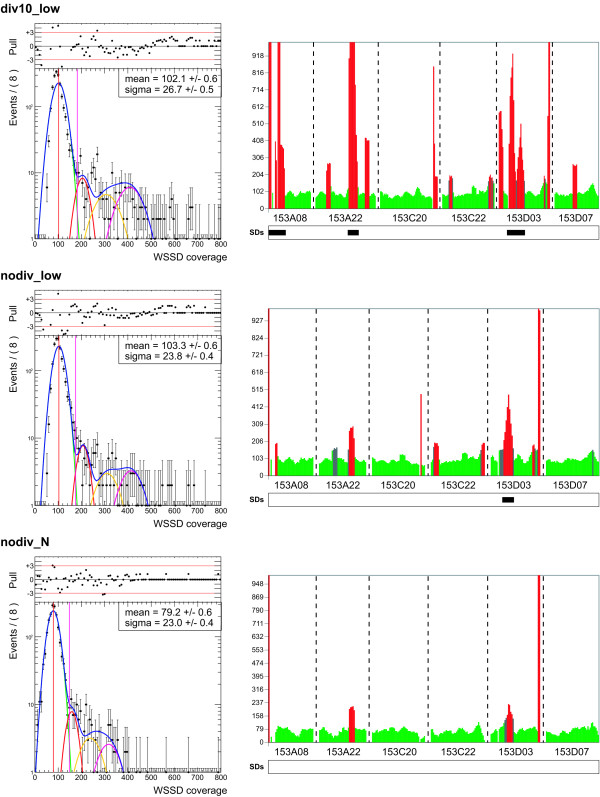
**Comparison of three repeat masking settings**. *Left panel *- The fit results of the WSSD coverage data sample from 39 single BAC-anchored loci (black dots) obtained using the "div10_low", "nodiv_low" and "nodiv_N" masking settings are displayed. The data are fitted to a model of four Gaussian distributions (G_1_, G_2_, G_3 _and G_4_) drawn in green, red, yellow and magenta, respectively, with their sum drawn in blue. Mean (red vertical line) and sigma (magenta vertical line) values in the box refer to Gaussian G_1_. On top, the normalized residuals distribution (Pull = (N_data_-N_fit_)/σ_data_, N and σ being the number of events and the error for each bin) is shown. When the pull distribution is fully between -3 and 3, fluctuations are only statistical. *Right panel *- WSSD outputs and segmentally duplicated regions according to the three masking methods of VV40024H153A08, 153A22, 153C20, 153C22, 153D03 and 153D07 BAC clones.

Of the 45 BAC clones tagged as single in FISH experiments, 39 were anchored to the *Vitis vinifera *genome [27]. We used the read depth over 5 kub windows in these 39 BAC-anchored loci to define the threshold to detect duplicated 5 kub windows for the three different masking settings discussed above. The resulting read depth distributions were similar (Figure 2). After testing some models (see Additional file 4), we choose to fit the data sample of single BAC 5 kub window coverages for each masking setting with a model made of four Gaussian distributions (G_1_, G_2_, G_3 _and G_4_) (Figure 2). G_2_, G_3 _and G_4 _curves had an average 2, 3 and 4 times the G_1 _average (avg) and a standard deviation √2, √3 and √4 times the G_1 _standard deviation (sd), respectively. We then considered as single the regions fitting in G_1 _and used the G_1 _average and standard deviation to calculate the threshold values for single and duplicated windows.

We established three categories for 5 kub windows based on their WSSD coverage or read depth: 1) read depth less than or equal to two sd above the avg (WSSD negative windows, green colored); 2) read depth greater than two sd above the avg and less than or equal to three sd above the avg (WSSD borderline windows, gray colored); and 3) read depth greater than three sd above the avg (WSSD positive windows, red colored) (Figure 1). We calculated the percentages of windows in the whole genome belonging to each category. We first considered all windows together, and then we divided them into five subgroups according to their masked sequence percentage: i) lower than 20%, ii) 20-40%, iii) 40-60%, iv) 60-80%, and v) greater than 80% (Additional file 5). We compared the occurrence of negative, borderline and positive windows in the full set and in the five subgroups, observing different distributions. It is noteworthy that for nodiv_low and nodiv_N methods, the higher the masked sequence percentage, the higher the percentage of WSSD positive windows. In the case of div10_low method, such a trend is valid for the first three subgroups, with almost half of the windows in the last three subgroups being duplication positive. These results showed that the percentage of WSSD positive windows generally increases with the increase of the window masking percentage, when comparing the window subclasses of the same method. This observation is true for all the three methods and is in agreement with previous works that demonstrated the relationship between segmental duplications and repetitive elements [28,29]. Moreover, the percentage of all WSSD positive windows goes down from 25 to 18 with decreased masking stringency: converting to N instead of lowercase and not limiting the allowed repeat divergence instead of limiting it to 10%.

We then compared the WSSD outputs of FISH-single BAC clones derived from the three methods (Figure 2). Several windows were positive in the div10_low method but negative in the nodiv_low and nodiv_N methods. Therefore, reads matching to unmasked repeats, which are more than 10% divergent from the consensus sequence, in the div10_low method widely determined the div10_low higher read depth. We observed a similar result when comparing the nodiv_low and nodiv_N methods because the lowercase repeat masked sequences still allowed matching reads, thus creating a higher depth of coverage. We then considered the nodiv_N method as the most suitable and appropriate masking setting to predict duplicated regions. This setting is better than the other two because it avoids the chance of false positives due to its lower masking stringency, not considering as duplicated the regions particularly rich in common repeats. Further, the resultant mean WSSD coverage value for windows in single copy (equal to 79.2) was perfectly consistent with the 12X coverage of the grapevine genome, as 735 (read size) × 79.2 (WSSD coverage) / 5000 (window size) = 11.6. A snapshot of WSSD coverages for all *Vitis vinifera *chromosomes is in the additional file 6.

In our subset of 100 random clones, 12 out of 17 (70.59%) clones classified as duplicated in FISH assays contained several WSSD positive regions (Additional file 7). The remaining five showed one to seven sequence gaps, reflecting the existence of a difficult-to-assemble genomic region, probably due to the presence of duplications. None of the FISH-duplicated clones were entirely WSSD positive. Of the single and undefined clones, 8/39 and 8/13, respectively, contained at least one WSSD positive region. Notably, the average number of gaps in the sequence assembly for FISH-duplicated clones is almost double the average in FISH-single clones (2.41 vs. 1.26), which stresses the difficulties in correctly assembling duplicated regions.

Further, we experimentally validated a subset of predicted duplicated regions. We selected 22 PN40024 BAC clones mapping to duplicated regions and used them as probes in FISH experiments. The results showed that 16/22 (73%) of the BAC clones were duplicated, four were single, one showed no result, and one was classified as undefined (data not shown). BAC VV40024H127M19 showed a tandem-duplication signal, whereas the others showed an interspersed pattern.

In conclusion, we estimated that SDs with > 94% sequence identity and >= 10 kb in length comprise 17.47% of the grapevine genome (85 Mb). We identified 2,642 duplicated intervals with a size mode equal to 20 kb and a maximum size of 379 kb (Additional files 8, 9).

As expected, contigs assigned to, but not placed on, chromosomes (random chromosomes) and contigs unassigned to any chromosome (unknown chromosome) are enriched for duplicated regions when compared to contigs assembled in chromosomes (nonrandom chromosomes) (27.64% and 20.57% vs. 16.76% of WSSD positive sequence) (Table 1). No chromosomes exceed the 38.48% (chr10_random) of duplicated fraction, except chr16_random (90.85%). Chromosome 16 shows the highest value for the percentage of duplicated sequence among the assembled nonrandom chromosomes (25.08%).

**Table 1:** Segmental duplications in the grapevine genome

chr	chr_size (bp)	dup_size (bp)	perc_dup	# dup intervals
chr1	23,037,639	2,733,492	11.87%	94
chr2	18,779,844	3,032,687	16.15%	104
chr3	19,341,862	3,860,663	19.96%	132
chr4	23,867,706	3,301,331	13.83%	118
chr5	25,021,643	3,688,952	14.74%	125
chr6	21,508,407	2,864,816	13.32%	89
chr7	21,026,613	2,334,595	11.10%	80
chr8	22,385,789	2,028,352	9.06%	86
chr9	23,006,712	5,694,956	24.75%	167
chr10	18,140,952	3,413,632	18.82%	111
chr11	19,818,926	2,155,608	10.88%	82
chr12	22,702,307	4,358,297	19.20%	143
chr13	24,396,255	4,592,630	18.83%	139
chr14	30,274,277	4,521,418	14.93%	159
chr15	20,304,914	4,479,245	22.06%	134
chr16	22,053,297	5,531,029	25.08%	149
chr17	17,126,926	2,453,620	14.33%	91
chr18	29,360,087	4,551,888	15.50%	147
chr19	24,021,853	5,810,980	24.19%	173
**Tot_nonrandom**	**426,176,009**	**71,408,191**	**16.76%**	**2,323**
chr1_random	568,933	136,397	23.97%	2
chr3_random	1,220,746	297,331	24.36%	9
chr4_random	76,237	0	0.00%	0
chr5_random	421,237	0	0.00%	0
chr7_random	1,447,032	140,206	9.69%	5
chr9_random	487,831	137,370	28.16%	4
chr10_random	789,605	303,818	38.48%	3
chr11_random	282,498	0	0.00%	0
chr12_random	1,566,225	403,711	25.78%	10
chr13_random	3,268,264	1,079,980	33.04%	28
chr16_random	740,079	672,372	90.85%	8
chr17_random	829,735	126,189	15.21%	4
chr18_random	5,170,003	1,365,584	26.41%	36
**Tot_random**	**16,868,425**	**4,662,958**	**27.64%**	**109**
**Tot_placed**	**443,044,434**	**76,071,149**	**17.17%**	**2,432**
chrUn	43,154,196	8,876,440	20.57%	210
**Tot_whole**	**486,198,630**	**84,947,589**	**17.47%**	**2,642**

Size and percentage of segmentally duplicated regions and the number of duplicated intervals for: each chromosome, all nonrandom chromosomes (Tot_nonrandom, from chr1 to chr19), all random chromosomes (Tot_random, from chr1_random to chr18_random), all nonrandom and random chromosomes (Tot_placed), and the whole genome (Tot_whole).

The grapevine genome and its duplicated portion are similar in their overall repeat and GC content (Additional file 10). Of note, SDs had a reduction of LINEs but enrichment in LTR elements, particularly Gypsy/DIRS1.

We searched for mitochondrial and plastid DNA sequences integrated in the grapevine nuclear genome by performing a BLAST search of the *Vitis vinifera *nuclear genome against *Vitis vinifera *organelle genomes as previously described [30,31]. We found that NUMTs (nuclear mitochondrial DNA) and NUPTs (nuclear plastid DNA) comprise 0.26% and 0.15%, respectively, of the grapevine genome (Table 2). The percentage of NUMTs reduces to 0.19% when we exclude chromosome "unknown" (chrUn). The grapevine nuclear genome shows NUMT content similar to that of *Arabidopsis thaliana*, representing the highest content among those known to date of sequenced plant genomes [30-32].

**Table 2:** Comparison of NUMT and NUPT content between grapevine whole-genome and segmentally duplicated regions

	Whole chromosome				Segmental duplications			
	
chr	NUMTs		NUPTs		NUMTs		NUPTs	
	
	bp	percentage	bp	percentage	bp	percentage	bp	percentage
chr1	35,148	0.15%	31,915	0.14%	7,942	0.29%	2,216	0.08%
chr2	23,352	0.12%	37,460	0.20%	5,029	0.17%	16,919	0.56%
chr3	46,530	0.24%	21,546	0.11%	3,746	0.10%	1,888	0.05%
chr4	40,855	0.17%	26,304	0.11%	8,651	0.26%	2,803	0.08%
chr5	36,974	0.15%	25,890	0.10%	6,936	0.19%	2,263	0.06%
chr6	42,918	0.20%	35,543	0.17%	14,186	0.50%	10,820	0.38%
chr7	29,780	0.14%	29,239	0.14%	1,282	0.05%	2,409	0.10%
chr8	52,538	0.23%	38,971	0.17%	16,355	0.81%	10,005	0.49%
chr9	40,017	0.17%	41,931	0.18%	12,529	0.22%	13,459	0.24%
chr10	40,034	0.22%	29,263	0.16%	6,255	0.18%	9,896	0.29%
chr11	32,762	0.17%	40,550	0.20%	8,227	0.38%	8,354	0.39%
chr12	79,290	0.35%	42,133	0.19%	46,728	1.07%	11,608	0.27%
chr13	59,784	0.25%	40,806	0.17%	22,390	0.49%	12,465	0.27%
chr14	59,292	0.20%	32,831	0.11%	12,094	0.27%	5,976	0.13%
chr15	25,964	0.13%	25,086	0.12%	5,899	0.13%	4,076	0.09%
chr16	55,547	0.25%	41,895	0.19%	21,245	0.38%	10,531	0.19%
chr17	26,574	0.16%	28,029	0.16%	3,702	0.15%	2,812	0.11%
chr18	60,444	0.21%	40,038	0.14%	22,523	0.49%	8,360	0.18%
chr19	39,838	0.17%	30,991	0.13%	12,876	0.22%	6,879	0.12%
**Tot_nonrandom**	827,641	**0.19%**	640,421	**0.15%**	238,595	**0.33%**	143,739	**0.20%**
chr1_random	1,763	0.31%	2,762	0.49%	78	0.06%	33	0.02%
chr3_random	872	0.07%	1,082	0.09%	0	0.00%	0	0.00%
chr4_random	0	0.00%	0	0.00%	-	-	-	-
chr5_random	642	0.15%	366	0.09%	-	-	-	-
chr7_random	2,355	0.16%	2,195	0.15%	352	0.25%	55	0.04%
chr9_random	525	0.11%	81	0.02%	93	0.07%	0	0.00%
chr10_random	111	0.01%	1,096	0.14%	0	0.00%	0	0.00%
chr11_random	667	0.24%	191	0.07%	-	-	-	-
chr12_random	1,522	0.10%	2,588	0.17%	674	0.17%	1,756	0.43%
chr13_random	8,607	0.26%	5,168	0.16%	4,914	0.46%	2,903	0.27%
chr16_random	636	0.09%	977	0.13%	636	0.09%	977	0.15%
chr17_random	94	0.01%	770	0.09%	0	0.00%	0	0.00%
chr18_random	5,302	0.10%	3,476	0.07%	2,709	0.20%	509	0.04%
**Tot_random**	23,096	**0.14%**	20,752	**0.12%**	9,456	**0.20%**	6,233	**0.13%**
**Tot_placed**	850,737	**0.19%**	661,173	**0.15%**	248,051	**0.33%**	149,972	**0.20%**
chrUn	417,301	0.97%	69,457	0.16%	159,039	1.79%	21,834	0.25%
**Tot_whole**	1,268,038	**0.26%**	730,630	**0.15%**	407,090	**0.48%**	171,806	**0.20%**

Interestingly, the grapevine genome and its duplicated portion exhibit different NUMT and NUPT content, either valuating sequences from nonrandom, random, placed or all chromosomes, with a remarkable increase in the percentage of NUMTs in the duplicated segments (Table 2). We found that 17% and 12% of duplicated intervals contain NUMTs and NUPTs, respectively.

SDs are depleted of genes, with a gene density almost half of the whole genome. 1,367/2,642 SDs (52%) entirely overlapped 2,589 predicted genes (9.83% of grapevine gene annotation), whereas 795 duplicated intervals (30%) did not contain any partial or entire gene (Table 3, Additional files 11, 12). However, these results may be affected by incomplete gene annotation, especially for SDs.

**Table 3:** Comparison of gene content between grapevine whole-genome and segmentally duplicated regions

chr	total genes	full dup genes	chr gene density	full dup gene density	ratio of densities
chr1	1,399	72	6.07	2.63	0.43
chr2	976	93	5.20	3.07	0.59
chr3	1,104	143	5.71	3.70	0.65
chr4	1,363	95	5.71	2.88	0.50
chr5	1,435	138	5.74	3.74	0.65
chr6	1,289	66	5.99	2.30	0.38
chr7	1,357	59	6.45	2.53	0.39
chr8	1,488	67	6.65	3.30	0.50
chr9	1,136	166	4.94	2.91	0.59
chr10	842	100	4.64	2.93	0.63
chr11	1,082	57	5.46	2.64	0.48
chr12	1,263	175	5.56	4.02	0.72
chr13	1,281	150	5.25	3.27	0.62
chr14	1,625	153	5.37	3.38	0.63
chr15	957	122	4.71	2.72	0.58
chr16	1,048	140	4.75	2.53	0.53
chr17	1,006	54	5.87	2.20	0.37
chr18	1,796	143	6.12	3.14	0.51
chr19	1,200	192	5.00	3.30	0.66
**Tot_nonrandom**	**23,647**	**2,185**	**5.55**	**3.06**	**0.55**
chr1_random	7	1	1.23	0.73	0.60
chr3_random	28	7	2.29	2.35	1.03
chr4_random	5	-	6.56	-	-
chr5_random	10	-	2.37	-	-
chr7_random	73	8	5.04	5.71	1.13
chr9_random	4	2	0.82	1.46	1.78
chr10_random	52	12	6.59	3.95	0.60
chr11_random	11	-	3.89	-	-
chr12_random	36	6	2.30	1.49	0.65
chr13_random	156	47	4.77	4.35	0.91
chr16_random	28	26	3.78	3.87	1.02
chr17_random	15	3	1.81	2.38	1.32
chr18_random	211	44	4.08	3.22	0.79
**Tot_random**	**636**	**156**	**3.77**	**3.35**	**0.89**
**Tot_placed**	**24,283**	**2,341**	**5.48**	**3.08**	**0.56**
chrUn	2,063	248	4.78	2.79	0.58
**Tot_whole**	**26,346**	**2,589**	**5.42**	**3.05**	**0.56**

Number of total genes (total genes), number of fully duplicated genes (full dup genes), chromosome gene density (chr gene density), density of fully duplicated genes in duplicated regions (full dup gene density), and ratio between full dup gene density and chr gene density (ratio of densities) are shown for each chromosome, all nonrandom chromosomes, all random chromosomes, all nonrandom and random chromosomes, and for the whole genome. Gene density is calculated as the average number of genes present in 100 kb of genomic sequence.

We searched the InterPro domain database to identify the functional motifs contained in *Vitis vinifera *annotated peptides and then compared functional motifs and biological functions between proteins encoded by unique and duplicated genes. We found that 43.73% of *Vitis vinifera *annotated proteins had no InterPro domain assignment, unlike 58.21% of the subset of proteins codified by genes entirely embedded in duplicated regions (Additional file 13). Twenty-six InterPro domains occurred only in duplicated genes, whereas 417 were enriched in duplicated versus single copy genes (Additional file 14). Most of the 26 domains are involved in respiratory and photosynthetic electron transport chains and in biosynthetic processes, such as of terpenoids and vitamin K. Among the InterPro domains with an enrichment factor greater than or equal to 14.47, corresponding to the 73 most enriched, several take part in translation (structural constituent of ribosome or involved in tRNA aminoacylation) or in biosynthetic processes, such as that of fatty acids and phenylpropanoids. In particular, the active sites of phenylalanine ammonia-lyase and chalcone/stilbene synthase, key enzymes in phenylpropanoid biosynthesis, were enriched in duplicated genes (found in 10 duplicated vs. 4 unique genes, and in 10 duplicated vs. 6 unique genes). Phenylpropanoids are secondary metabolites important for normal growth and in responses to environmental stress and include flavonoids, stilbenoids, phytoalexins and cell wall components. They provide protection from ultraviolet light, defend against herbivores and pathogens, and mediate plant-pollinator interactions such as floral pigments and scent compounds. Other examples of enriched domains are ferritin, which is involved in iron storage, and annexin, present in a family of calcium-dependent phospholipid-binding proteins [33] involved in inhibition of phospholipase activity, exocytosis and endoctyosis, signal transduction, organization of the extracellular matrix, resistance to reactive oxygen species and DNA replication [34].

We then analyzed InterPro domains contained in the 100 most duplicated genes (genes embedded in regions with the highest read depth values) (Additional file 15). Several genes among the 100 most duplicated contain functional domains with oxidoreductase activity, such as ferrodoxin, enzymes involved in the respiratory and photosynthetic electron transport chains, aldo-keto reductase, glucose/ribitol dehydrogenase, and stearoyl-acyl-carrier-protein desaturase. The cytochrome P450 domain, present in a superfamily of heme-containing mono-oxygenases and important in plants for the biosynthesis of several compounds such as hormones, defensive compounds and fatty acids, was frequent. Several genes that might be involved in the regulation of transcription are listed: they codify for the SET domain, found in hystone lysine methyltransferases [35], or for the homeodomain. Genes encoding proteins containing tetratrico peptide repeats, which mediate protein-protein interactions, or pentatrico peptide repeats, which are thought to mediate RNA-binding, are also present (Additional file 15).

Among the 100 most duplicated genes, 21 detect homologous genes in mitochondria and/or chloroplast genomes. Excluding genes in chrUn that might contain segments erroneously assigned to the nuclear genome, these data remain valid though less pronounced (data not shown).

## Discussion

This is the first genome-wide analysis quantifying duplicated loci and genes as well as mitochondrial and chloroplast DNA sequences in the nuclear genome of grapevine. Our results have revealed several interesting features of SDs and the genome organization in *Vitis vinifera *that were not previously characterized.

This study used a two-pronged approach: molecular cytogenetics and *in silico *analysis to discern single and duplicated regions in the *Vitis vinifera *genome. The quality of common repeat annotation impacts the assessment of the SD content in a genome. Most recent WSSD analyses completed on chimpanzee, dog and bovine genomes masked to lowercase repeats having a divergence < 10% from the repeat consensus [24-26] to more precisely calculate read depth values. Our comparison of repeat masking methods revealed that more stringent masking criteria should be avoided in the case of grapevine. We integrated and resolved two limits in the SD analysis: the lack of resolution in detecting small duplications using FISH and the existence of highly divergent repetitive elements in the grapevine genome. In this work, we suggest a way to overcome these limits and determine the coverage threshold for duplications, combining the selection of single regions by FISH with a statistical analysis of WSSD coverage values.

The French-Italian Public Consortium for Grapevine Genome Characterization reports that 41.4% of the grapevine genome (8X release) is composed of repetitive/transposable elements [4]. This percentage derives from the integration of different approaches to identify the repetitive elements: the frequency of manually annotated transposable elements is 17.47%, whereas the frequency of ReAS derived "repetitive sequences" is 38.81%, as reported in Table S7 of the work [4].

We found that 24.24% of the grapevine genome (12X release) is composed of interspersed repeats using the RepBase library of *Vitis *repeats (Additional file 10). This frequency totally agrees with the one previously reported for the heterozygous grapevine variety (10.7X, 21% of interspersed repeats) [22].

The ReAS algorithm allows the identification of transposable elements using the unassembled reads of WGS [36], but its results are biased by a 55% of probable artifacts, in part due to the accounting of high copy number SDs. Since in this work we searched for SDs, we preferred to mask the grapevine genome using only manually annotated transposable elements, to avoid the occurrence of high number of false negatives. Nevertheless, it should taken into account that our analysis might comprise false positives, due to unannotated transposable elements, that the ReAS software is able to detect. Our choice was driven by the preference of getting some false positive duplications, which are actually transposable elements, instead of losing most real segmental duplications (false negatives).

We focused on highly similar SDs to identify recently duplicated regions in the grapevine genome thus representing candidate hot spots for *de novo *duplication and/or copy number variation among existing grapevine varieties. According to the remarkable content of highly similar Pinot Noir SDs, we speculate that some of these regions underlie the molecular basis of some phenotypical features, and the copy number variation of genes under investigation should be considered in future studies aimed at the identification of genetic differences among grapevine varieties. A noteworthy observation from our analysis is the high content of organelle DNA (NUMTs and NUPTs) as part of Pinot Noir duplications repertoire suggesting that organelle DNA sequence integration, other than SD events, played an important role in grapevine nuclear genome evolution.

Two alternative models have been proposed for the evolution of the grapevine genome. They both derive from the primary observation that many regions of the genome appear in triplicate. Jaillon et al. [4] suggest that the grapevine genome originated from the contribution of three ancestral genomes (paleo-hexaploid organism). Alternatively, Velasco et al. [22] suggest a whole-genome duplication event shared by all dicotyledons followed by a large-scale duplication event, likely a hybridization event, in the *Vitis *lineage in close proximity to the *Vitis *speciation event. Both these models assume the occurrence of large-scale duplication events during *Vitis *evolution. The duplicative events we tracked in our map are quite recent (> 94% identity) and cannot be used to trace any long-range evolutionary history of the *Vitis *genome.

Recent genomic sequence data provide substantial evidence for the abundance of duplicated genes in all organisms surveyed [37-43]. Many studies have described the involvement of SDs in gene evolution. Several functional categories are enriched among these genes, including immune response, xenobiotic recognition, reproduction and nuclear functions. This suggests an important role for SDs in adaptive evolution: they might have facilitated adaptation to changes especially when a diversity of responses was advantageous [14,41,42,44-47].

In this study, we characterized which genes have been preferentially duplicated in the grapevine genome, likely giving rise to novel gene families. We performed a genome-wide comparative analysis of functional domains traced in single versus duplicated genes and also focused on the 100 most duplicated genes, which revealed two important aspects. First, duplicated genes are enriched for genes without annotated functional domains (58.21% of duplicated genes vs. 43.73% of the whole genome). Second, duplicated genes show some functional biases. A few genes coding for the cytochrome P450 domain, found in plant enzymes involved in the biosynthesis of several compounds such as hormones, defensive compounds and fatty acids [48,49], are among the 100 most duplicated. Further, the active sites of phenylalanine ammonia-lyase and chalcone/stilbene synthase, key enzymes in phenylpropanoid biosynthesis, were enriched in duplicated genes. Previous grapevine genome sequencing projects already highlighted the existence of several copies of genes encoding these enzymes [4,22]. Stilbene synthase catalyzes the synthesis of resveratrol, the major compound responsible of cardioprotective abilities of grapes and wine, attenuating atherosclerosis and ischemic heart [50,51]. Additional duplicated genes involved in the biosynthesis of terpenoids and vitamin K have an impact in human health [52,53]. These data suggest a preferential expansion through duplication of genes involved in responses to environmental stress [54-56]. The duplication of these genes might improve not only the plant resistance against biotic and abiotic stresses, but also the nutritional value of grapes and grape products for human consumption. According to our data, in *Vitis vinifera*, like in humans and mammals, most duplicated genes are responsible for adaptation or response to environmental changes and thus are strongly relevant for cultural needs, where the protection of plants from pathogens and climate variability is of great importance. However, since we have not further investigated the fate of the duplicated genes, they could comprise both pseudogenes and novel genes. The analysis of the evolutionary fate of the identified genes and whether they experienced neofunctionalization, subfunctionalization, conservation of function, or nonfunctionalization [57] required further specific and targeted studies on specific gene sequences, at the moment not available. Our genome-wide approach has defined an SD map of the grapevine genome and points out SD regions on which to focus future studies aimed at characterizing embedded coding sequences.

The enrichment of InterPro domains involved in respiratory and photosynthetic electron transport chains in duplicated vs. unique genes, as well as the role of the highest copy number genes in these two processes, was striking. As these genes are located in nuclear, mitochondrial and chloroplast genomes, their duplication extent may be due to a considerable process of transfer of organellar DNA to the nucleus in *Vitis vinifera*, or to the preferential duplication of such sequences in the nuclear genome, after their movement from the organelle genomes.

## Conclusions

The grapevine represents one of the earliest domesticated fruit crops and, since antiquity, has been cultivated for consumption of its fruit or producing wine. Genetic information, such as linkage maps, genome-wide association studies, and genome selection, is increasingly being used to guide breeding efforts in grapevines. All of these approaches are focused to isolate varieties showing specific characteristics used for cultural needs but in a time-consuming way. In our genome-wide study, we analyzed and identified candidate regions and genes embedded in SDs as possible targets to improve existing grapevine varieties. Our SD map represents a useful tool for future comparative studies to other grapevine varieties to identify common or distinctive traits with the aim of selecting the ideal variety for cultural needs. Targeted approaches to increase the amount or expression of these genes would be critically important to further improve and use grapes as great source of essential substances for human health.

## Methods

### FISH experiments

The grapevine (*Vitis vinifera*) Pinot Noir canes came from certified mother vine of Vivai Cooperativi Rauscendo. The canes were stored at 4°C and 90% relative humidity and cut into approximately 20 cm pieces. Cuttings were washed in 3% bleach and immersed 2 cm in SPRINTEX NEW L. 5 mL/L solution for 1 h. Cuttings were then washed and kept in water until they germinated.

Interphase nuclei and metaphase spreads were obtained using a drop-spreading technique. We used the method described by Kesara Anamthawat-Jonsson [58] with the following modifications. Actively growing leaf buds were treated in 2 mM 8-hydroxyquinoline for 2 h at room temperature, then 2 h at 4°C to accumulate metaphases. Leaf buds were fixed, rinsed with distilled water, and digested for 5 h in the enzyme mixture (75 mM KCl, 7.5 mM EDTA, 2.5% Pectinase and 2.5% Cellulase). The protoplasts were isolated by filtering the suspension through a nylon mesh of 100 μm. 12 ml of 75 mM KCl were added to the protoplast suspension and incubated for 15 min. The suspension was centrifuged at 4500 g for 5 min, the supernatant was discarded, and 8 mL of fixative (methanol-acetic acid 3:1) were added to the protoplast pellet. The suspension was left at 4°C overnight. The next day, the fixative was changed twice. The protoplast pellet was diluted in fixative at a proper concentration and protoplasts were dropped on slides.

FISH probes were derived from *Vitis vinifera *Pinot Noir 40024 BAC library, which was developed by INRA-CNRGV [59], Genoscope [60] and URGV [61,62].

Slide treatment and FISH hybridization were performed as previously described. Briefly, BAC probes were directly labeled with Cy3-dUTP by nick-translation. Slides and probes were denatured at 75°C for 2 min. Hybridization was performed at 37°C overnight in 2X SSC (sodium chloride and sodium citrate), 50% formamide, 10% dextran sulfate, 3 μg of *Vitis vinifera *C0t-1 and 5 μg of sonicated salmon sperm DNA. High stringency, post-hybridization washing was at 60°C in 0.1X SSC, three times. *Vitis vinifera *C0t-1 was prepared [63] from *Vitis vinifera *Pinot Noir genomic DNA extracted from leaves [64,65]. Digital images were obtained using a Leica DMRXA epifluorescence microscope equipped with a cooled CCD camera.

### Data sets

*Vitis vinifera *chromosome, mRNA and peptide sequences were downloaded from the GENOSCOPE data repository site [66]. The chromosome sequences were assembled by GENOSCOPE, CRIBI (Consortium VIGNA) and IGA and released in March 2010 (12X WGS coverage). We obtained *Vitis vinifera *WGS reads and related clip (sequence quality data) files from the NCBI Trace archive [67]. 8,743,362 WGS reads were available when we started the analysis (of these, 77,237 items were BAC end sequences). The genomic location and size of BAC clones were obtained from the URGI *Vitis vinifera *genome browser [27].

### WSSD computational analysis

We discarded 110,537 reads according to these assessments: 1) low quality and/or contamination evaluation in clip file; 2) percent errors for the clipped trace greater than 6.00; and 3) length of the high-quality read portion smaller than 300 bp. We clipped the remaining 8,632,825 reads (98.7%); the average sequence size was ~735 bp (range 300-1,447 bp), thus the final estimated coverage of the genome was 13X.

We masked the chromosome sequences using both RepeatMasker [68] (with the option -species "vitis vinifera") and Tandem Repeats Finder [69] (parameters: match 2, mismatch 7, indels 7, PM 80, PI 10, minscore 50, maxperiod 500). We defined the limits of a series of non-overlapping sequence windows. Each window contained exactly one thousand of unmasked bases (1 kub). If a window included a sequence gap, the window was discarded and the first limit of a new one was picked at the first unmasked nucleotide after the gap.

We performed a megablast (version 2.2.23 [70]) alignment of the clipped *Vitis vinifera *reads to the repeat masked *Vitis vinifera *genome, with the following parameters: -D 3 -p 93 -U T -F m -s 220. We parsed the megablast output to select only matches larger than 300 bp. In the case of conversion to lowercase of the masked nucleotides, we further selected only the alignments with less than 200 bp within a masked sequence. Then we counted the number of remaining matches that fell in the previously defined 1 kub windows (we considered the middle nucleotide of each match). As in previous work, we defined a class of 5 kub windows merging five 1 kub contiguous windows, and sliding of a 1 kub window to define the next one. If a sequence gap was encountered, the sliding was interrupted and the next 5 kub window obtained from the five contiguous 1 kub windows after the gap. In this way, we obtained the counts for a series of 5 kub windows, most of which overlapped to the two neighbors of 4 kub. We will refer to these counts as WSSD coverage values or read depth over 5 kub windows.

We selected a list of BACs from FISH assays designated as single. BAC clones were anchored in the grapevine genome assembly through BAC end mapping provided by the Istituto di Genomica Applicata at the URGI *Vitis vinifera *genome browser [27]. We extracted the WSSD coverage values belonging to the corresponding genomic regions and performed statistical analyses. The average and standard deviation of Gaussian G_1 _(see next paragraph) were used to set the threshold for duplication detection. All intervals having at least six out of seven consecutive windows with a significantly higher depth of coverage (number of reads greater than or equal to the average plus 3 standard deviations) were considered SDs. Contiguous intervals were then merged, and the average WSSD coverage for 5 kub windows was calculated for each region. To validate the prediction of duplicated regions, we randomly selected 22 BAC clones mapping in duplicated intervals greater than 90 kb and tested them by FISH. Circular graphs of the WSSD coverages of *Vitis vinifera *chromosomes were produced using the Circos tool [71].

It should be taken into account that some discrepancies between *in silico *predictions and experimental data may be derived from the different biological sources of Pinot Noir sequenced genomic DNA and cytogenetic specimen.

### Statistical analysis of WSSD coverage values

We fitted the WSSD coverage values of windows from 39 single BAC-anchored loci using the RooFit tool of the software ROOT [72] with a model of N = 4 Gaussian distributions. The probability density function (PDF) used to describe the distribution of the WSSD coverage values is defined as follows:

$$PDF=\overset{4}{\underset{n=1}{\sum }}f_{n}G_{n}\left(avg\cdot n;sd\cdot \sqrt{n}\right),\text{with}\overset{4}{\underset{n=1}{\sum }}f_{n}=1$$

where avg and sd equal the average and standard deviation values of Gaussian G_1 _(n = 1). This model arranges average and standard deviation values of the following Gaussians as related to the values of the first one (G_1_): avg_Gn _= avg_G1_*n, sd_Gn _= sd_G1_*√n. The sum of the areas under all Gaussian curves is required to equal 1.

In order to retrieve the best fit to the distribution of the WSSD coverages, a maximum likelihood fit was done. The fit program evaluates the likelihood L comparing the PDF above to the data set. The parameters of the best fit are retrieved by the minimization of the function -logL, performed by the MINUIT software [73].

### Gene content analysis

We took advantage of genes already mapped to the reference genome sequence of *Vitis vinifera *and considered genes to be duplicated if they mapped entirely within the coordinates of predicted duplicated regions. We then compared the gene density of each chromosome with the one of its segmentally duplicated fraction. Gene density was calculated as the average number of genes present in 100 kb of genomic sequence.

We searched PROSITE, PRINTS, ProDom, SMART, Tigr, SUPERFAMILY, Gene3D, PANTHER and HAMAP databases for InterPro domains present in grapevine annotated peptide sequences and in grapevine mitochondrial and plastid peptide sequences [74]. We retained true positive matches (status = T) and removed those with no InterPro number assignment (NULL tagged). We then calculated an enrichment factor for each InterPro domain found in grapevine peptides encoded by the nuclear genome. The enrichment factor was defined as the ratio between the fraction of the InterPro domain among all the domains found in duplicated peptides and the fraction of the same domain among all the domains found in unique peptides. Additionally, we selected the 100 most duplicated genes according to the average of 5 kub window coverages of the duplicated merged intervals and identified the matched InterPro domains for these genes. We identified the InterPro domains shared by proteins encoded by nuclear and organelle genes.

### NUMT and NUPT analysis

*Vitis vinifera *full-length organellar nucleotide sequences (NC_012119.1 and NC_007957.1) were retrieved from NCBI [75] and masked using RepeatMasker [68] (option -species "vitis vinifera"). Then we locally performed BLASTN alignments (blast 2.2.23 [70], standard settings and threshold of 10^-4^) of masked grapevine mitochondrion and chloroplast genomes to the grapevine nuclear genome, SDs and the 100 most duplicated mRNA sequences to identify genes deriving from organelle genomes. We calculated the percentage of NUMTs and NUPTs in the whole genome and SDs, counting only once the nucleotides in the genome that have more than one BLAST hit to mitochondria or plastid sequences.

## List of abbreviations

avg: average; BAC: bacterial artificial chromosome; BES: BAC end sequences; BLAST: basic local alignment search tool; chr: chromosome; FISH: fluorescence *in situ *hybridization; kub: kilo of unmasked bases; LINE: long interspersed nuclear element; LTR: long terminal repeats; NUMT: nuclear mitochondrial DNA; NUPT: nuclear plastid DNA; OEA: one-end anchored; PDF: probability density function; sd: standard deviation; SD: segmental duplication; SSC: sodium chloride and sodium citrate; WGS: whole-genome shotgun; WSSD: whole-genome shotgun sequence detection.

## Authors' contributions

MV and DA conceived the study. GG, MG, and FNC carried out the FISH experiments. PD performed the computational analysis. MM performed the statistical analysis and contributed to manuscript writing. GG, PD, MV and DA participated in the design of the study, analyzed data and drafted the manuscript. All authors read and approved the final manuscript.

## Supplementary Material

Additional file 1 **FISH results of a tandem-duplicated clone**. FISH signals on grapevine metaphase chromosomes and interphase nucleus of the VV40024H153B02 tandem-duplicated BAC clone. Click here for file

Additional file 2 **FISH results of 100 random clones from the VVPN40024 library**. The table lists the clone ID, chromosome mapping and FISH result of 100 random BAC clones from the VVPN40024 library. Mapping information on 12X grapevine genome assembly is obtained from http://urgi.versailles.inra.fr/cgi-bin/gbrowse/vitis_12x_pub/. OEA, one-end anchored. Click here for file

Additional file 3 **Identity percentages between end sequences of the tandem-duplicated BACs**. Identity percentages between end sequences of the five BAC clones from plate 153 showing the 4-cluster pattern in FISH experiments. Click here for file

Additional file 4 **Supplemental Note**. The note reports the test of five different statistical models to identify the most appropriate one describing the WSSD coverage data. Click here for file

Additional file 5 **Comparison of three repeat masking settings**. The table shows the percentages of WSSD-negative (green), borderline (gray) and positive (red) 5 kub windows among all windows in the grapevine genome and in five subgroups, defined by the percentage of masked sequence in the window. The percentages are given for the three repeat masking settings "div10_low", "nodiv_low" and "nodiv_N". Click here for file

Additional file 6 **WSSD coverage of *Vitis vinifera *chromosomes**. The graphs illustrate the WSSD coverage of all *Vitis vinifera *chromosomes and were produced using the Circos tool. WSSD negative, borderline and positive windows are represented by green, gray and red colored bars, respectively. Last segment of chrUn sequence misses WSSD coverage values as it is composed of blocks, spaced out by gaps, too short to calculate the WSSD coverage on 5 kub windows. Click here for file

Additional file 7 **Segmental duplication data of single, undefined and duplicated clones**. The table lists the number and percentage of clones containing segmental duplications, and the average and standard deviation values of gap number, for single, undefined and duplicated clones designated according to FISH results. Click here for file

Additional file 8 **Distribution size of duplicated intervals**. The graph shows the number of duplicated intervals according to their size. Click here for file

Additional file 9 **Duplicated intervals identified in the *Vitis vinifera *PN40024 genome**. The table lists all the duplicated intervals identified in the *Vitis vinifera *PN40024 genome and reports their chromosome position, size and average WSSD coverage. Click here for file

Additional file 10 **GC and repeat content of the whole-genome and segmentally duplicated regions**. The table compares the GC level and repeat content defined by the RepeatMasker tool between grapevine whole-genome and the identified segmentally duplicated regions. Click here for file

Additional file 11 **Fully and partially duplicated gene content in grapevine chromosomes**. The table lists the number of total genes, number of fully and partially duplicated genes (full and part dup genes), chromosome gene density, density of fully and partially duplicated genes in duplicated regions (full and part dup gene density), and ratio between full and part dup gene density and chr gene density (ratio of densities), for each chromosome, all nonrandom chromosomes, all random chromosomes, all nonrandom and random chromosomes, and for the whole genome. Gene density is calculated as the average number of genes present in 100 kb of genomic sequence. Click here for file

Additional file 12 **Gene-containing duplicated intervals in grapevine chromosomes**. The table lists the number and fraction of duplicated intervals with fully duplicated genes and with fully and/or partially duplicated genes, in each grapevine chromosome, all nonrandom chromosomes, all random chromosomes, all nonrandom and random chromosomes, and in the whole genome. Click here for file

Additional file 13 **Summary of InterPro scan results for the whole genome and its segmentally duplicated portion**. The table lists the number of genes, number of InterPro domains, number of InterProScan matches, number of genes with at least one InterPro domain, number of genes with no InterPro domain, percentage of genes with at least one InterPro domain, percentage of genes with no InterPro domain, for the whole genome and its segmentally duplicated portion. Click here for file

Additional file 14 **InterPro domains identified in grapevine proteins and their enrichment factor in duplicated vs. unique genes**. The table lists the InterPro domains identified in proteins codified by the grapevine genome, their occurrence in unique and duplicated genes, and their enrichment factor in duplicated vs. unique genes. The InterPro domains are sorted by their enrichment factor in descending order. Click here for file

Additional file 15 **Top 100 duplicated genes**. The table lists the 100 genes embedded in regions with the highest read depth values. It reports their chromosome location, the mean WSSD coverage of the region and their InterPro domain content. Further, the table indicates whether the genes detect homologous genes in chloroplast and/or mitochondria genomes. Click here for file
