# Supplementary figures and images for: Analysis of high-identity segmental duplications in the grapevine genome
Source: BMC Genomics. 2011 Aug 26;12:436. doi: 10.1186/1471-2164-12-436 (PMC3179966; doi:10.1186/1471-2164-12-436)

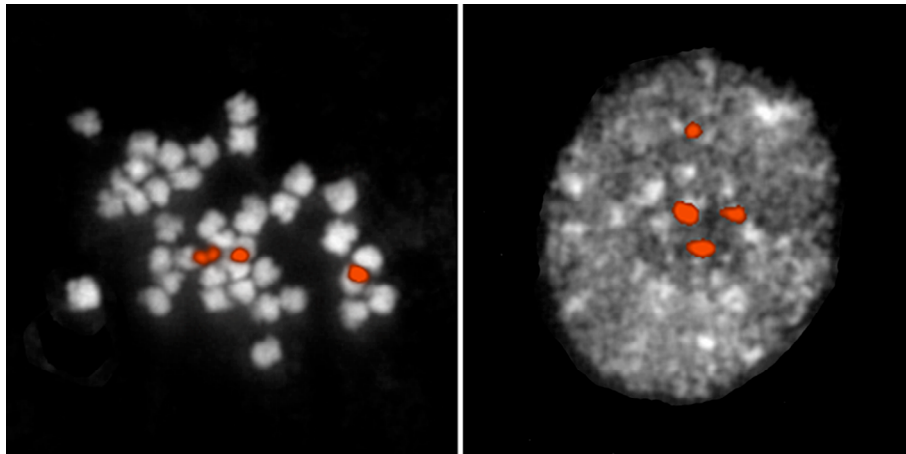

**FISH results of a tandem-duplicated clone.**

Supplement: Additional file 1 — FISH results of a tandem-duplicated clone. FISH signals on grapevine metaphase chromosomes and interphase nucleus of the VV40024H153B02 tandem-duplicated BAC clone. [file 1471-2164-12-436-S1.PDF]

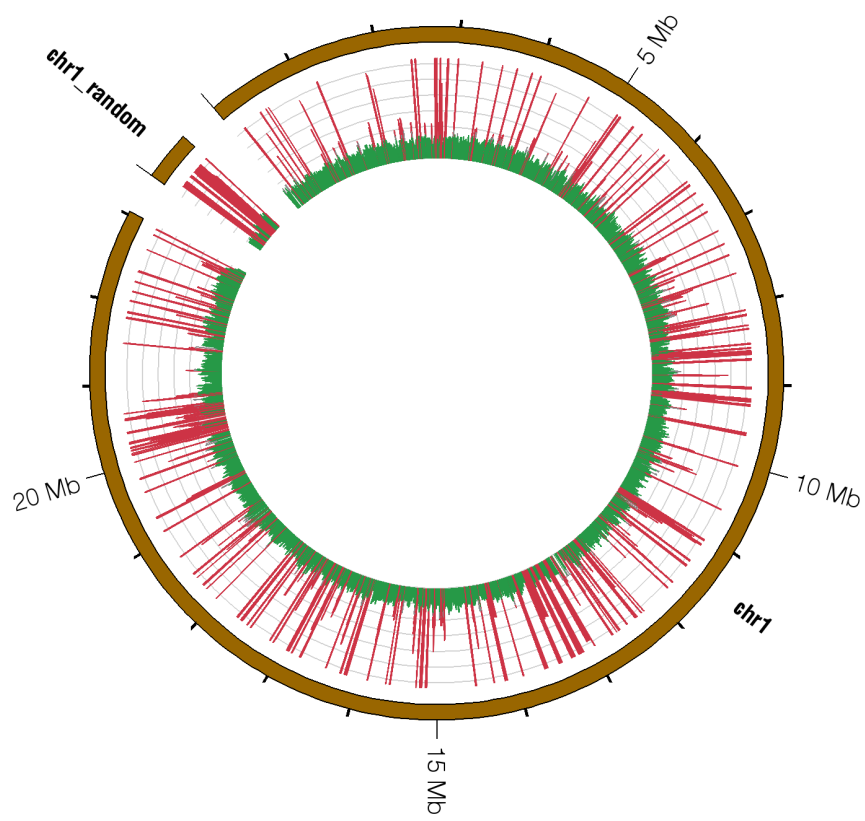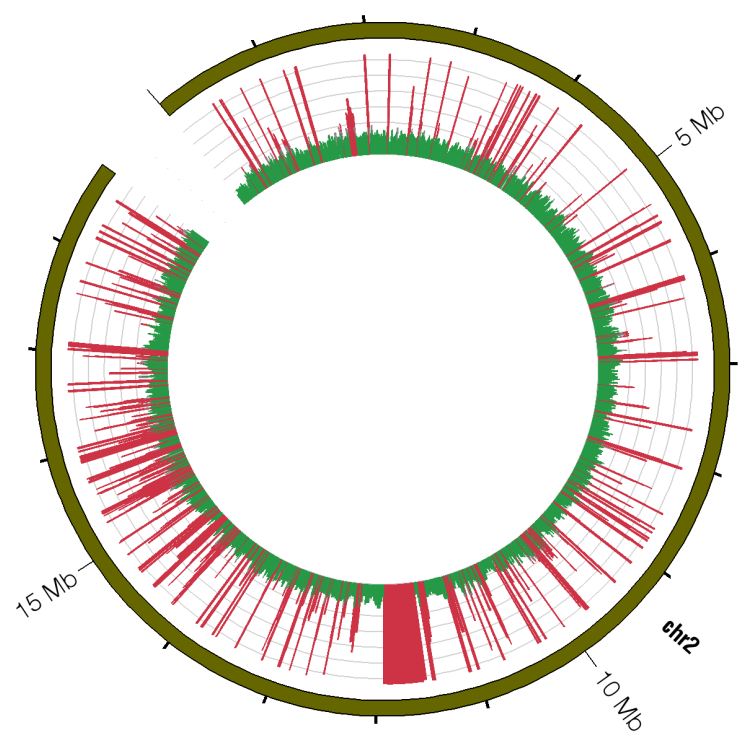

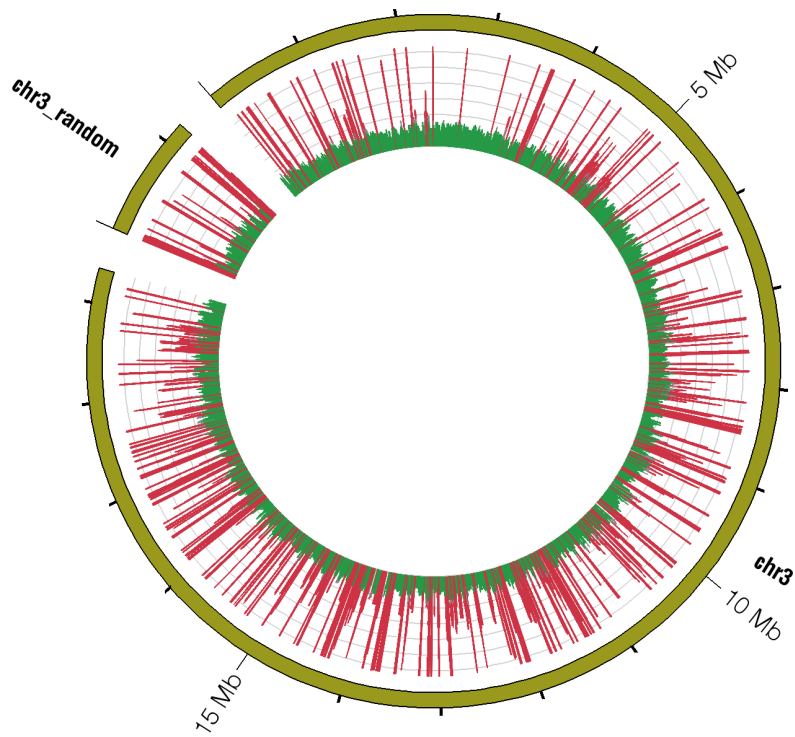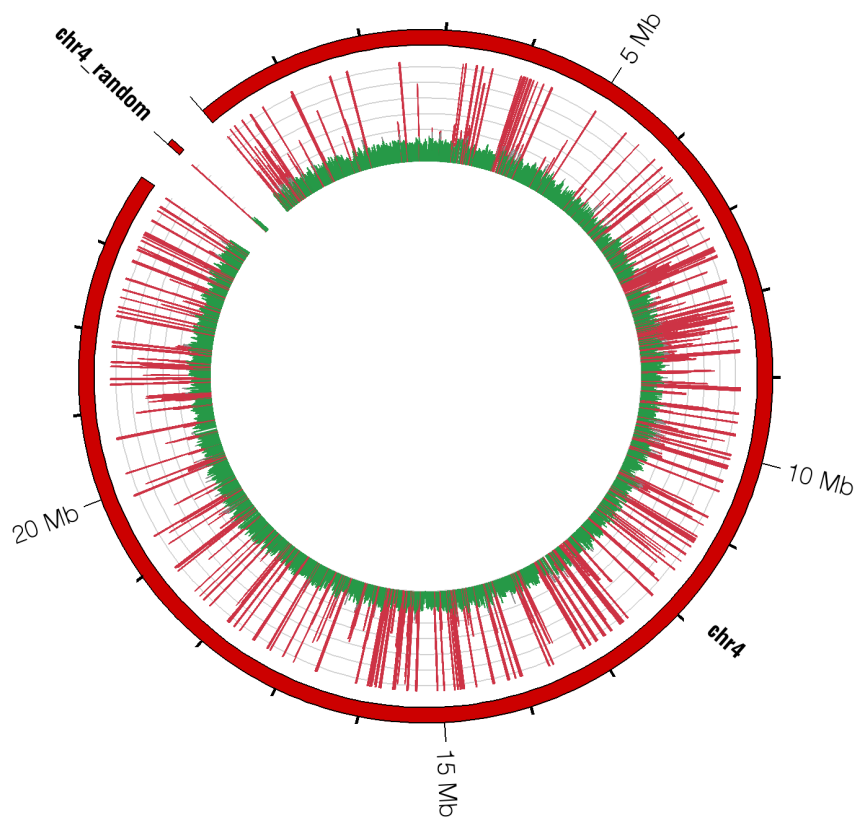

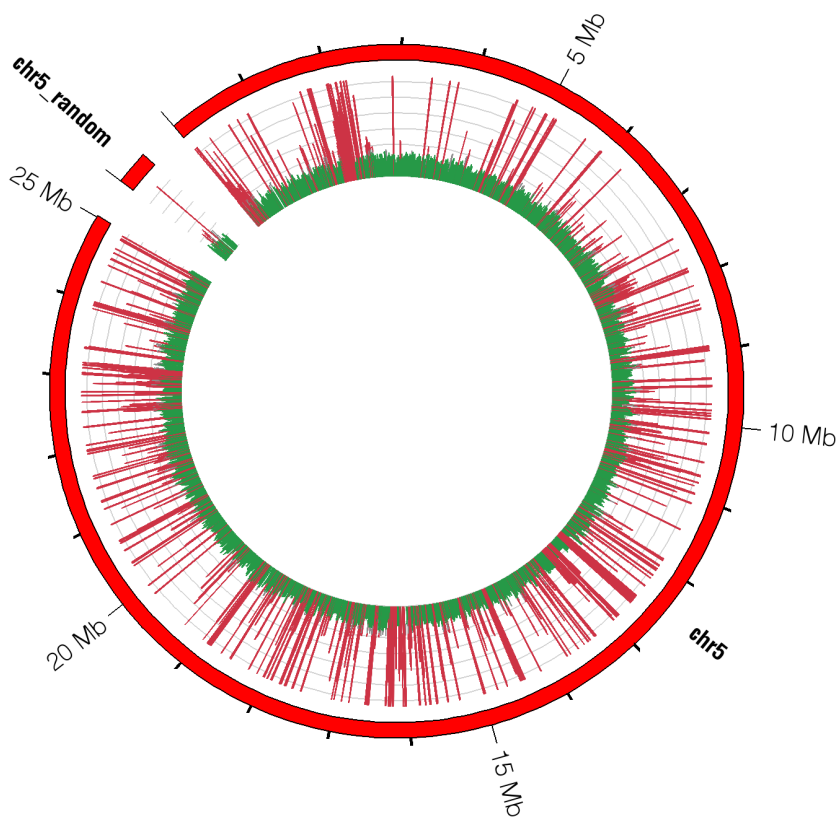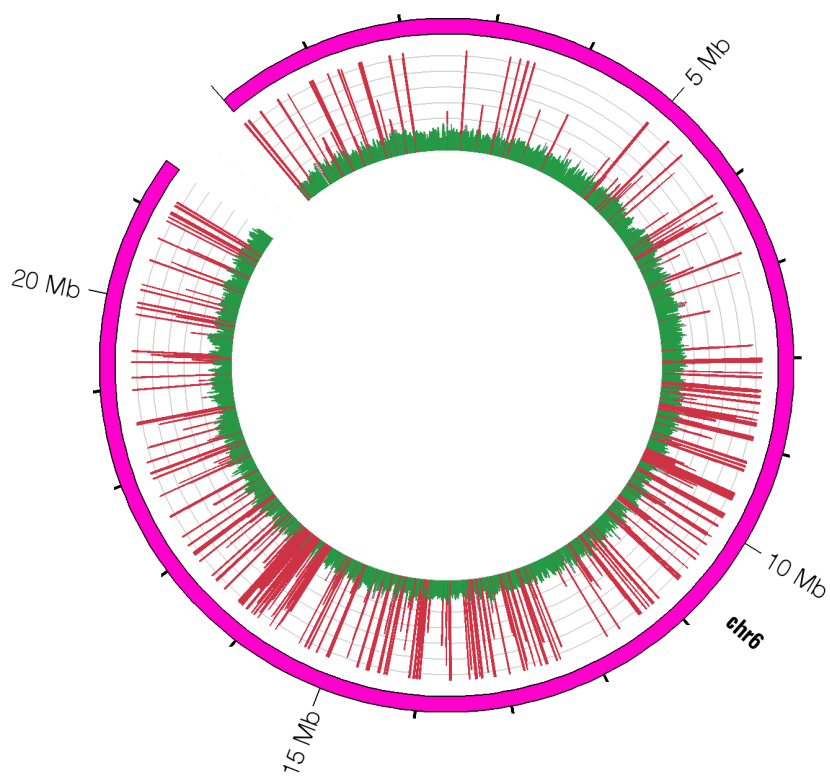

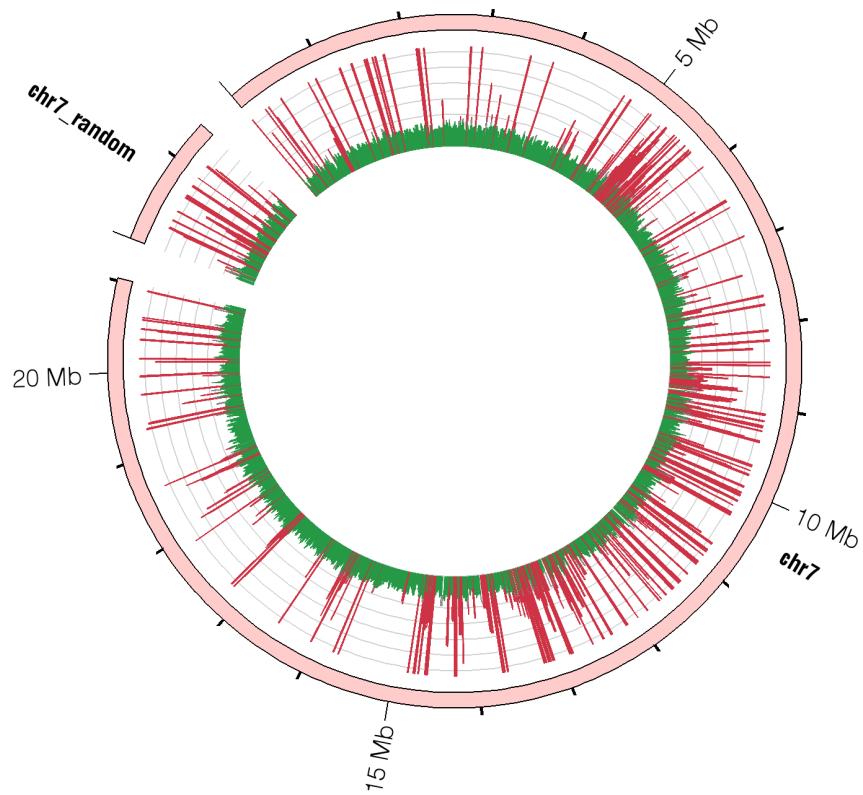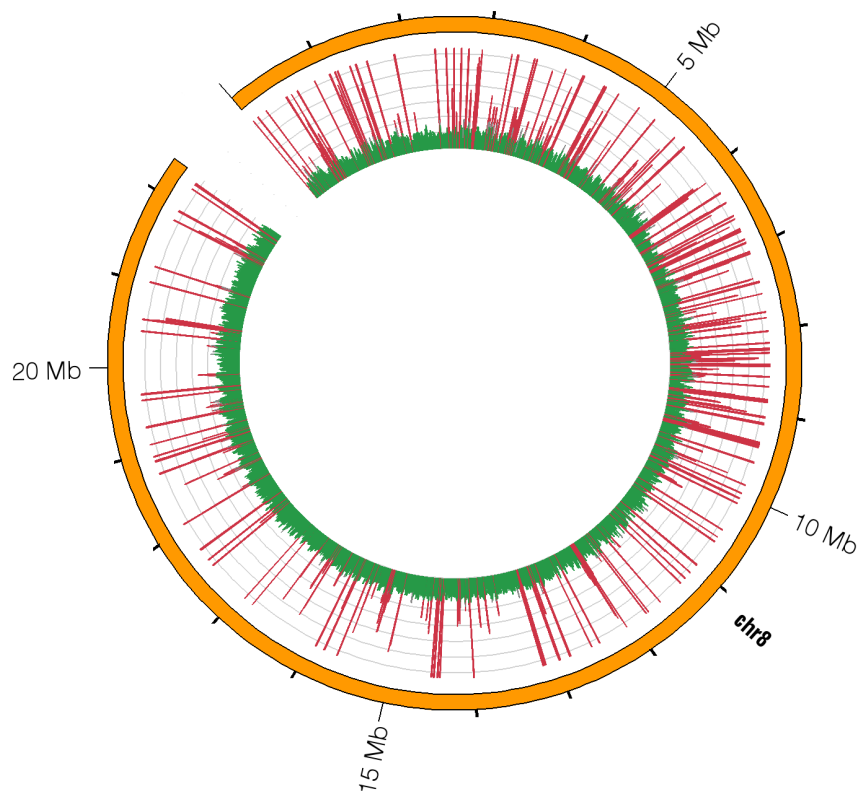

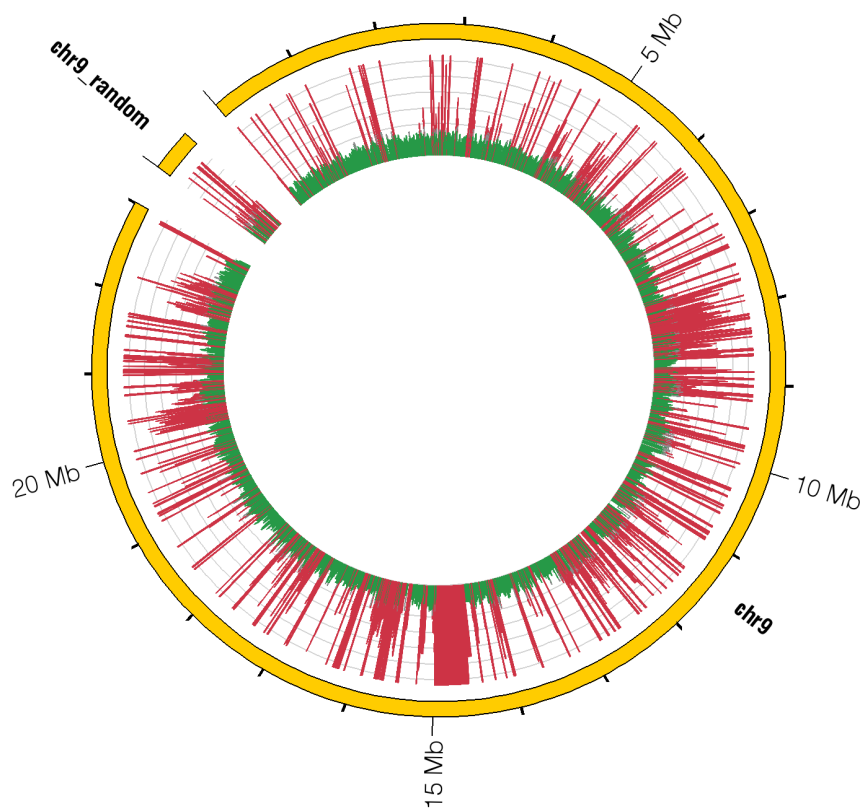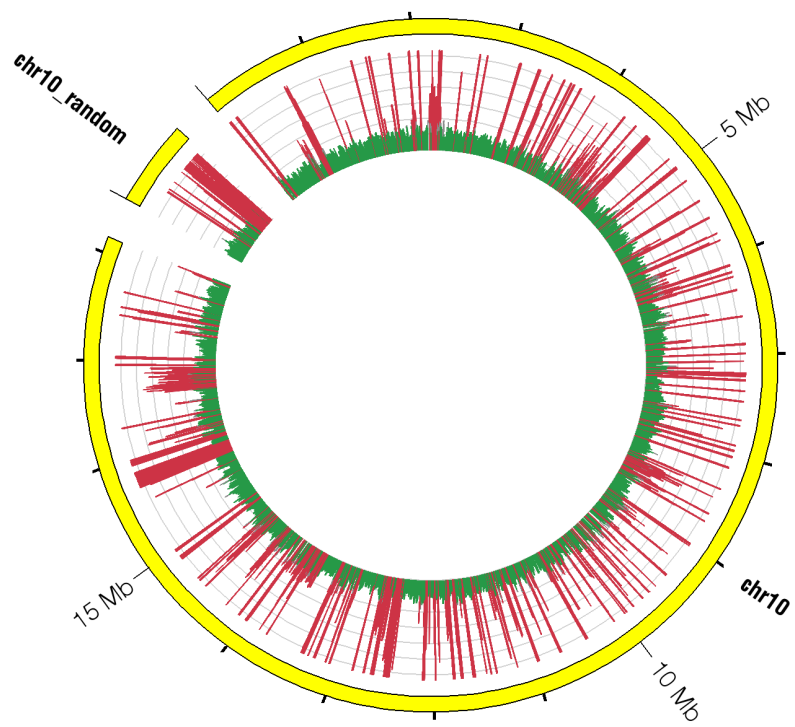

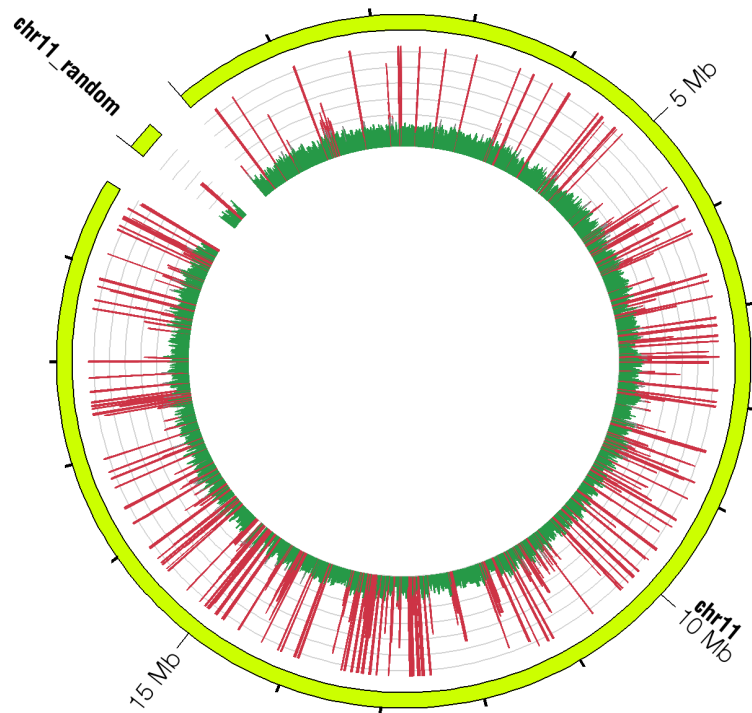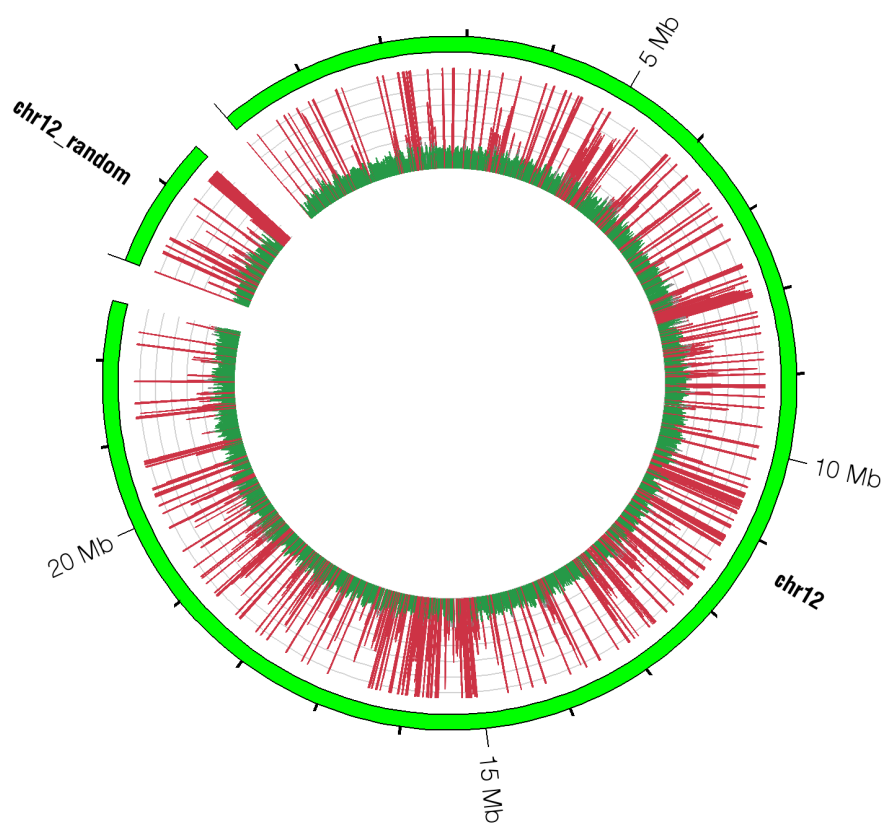

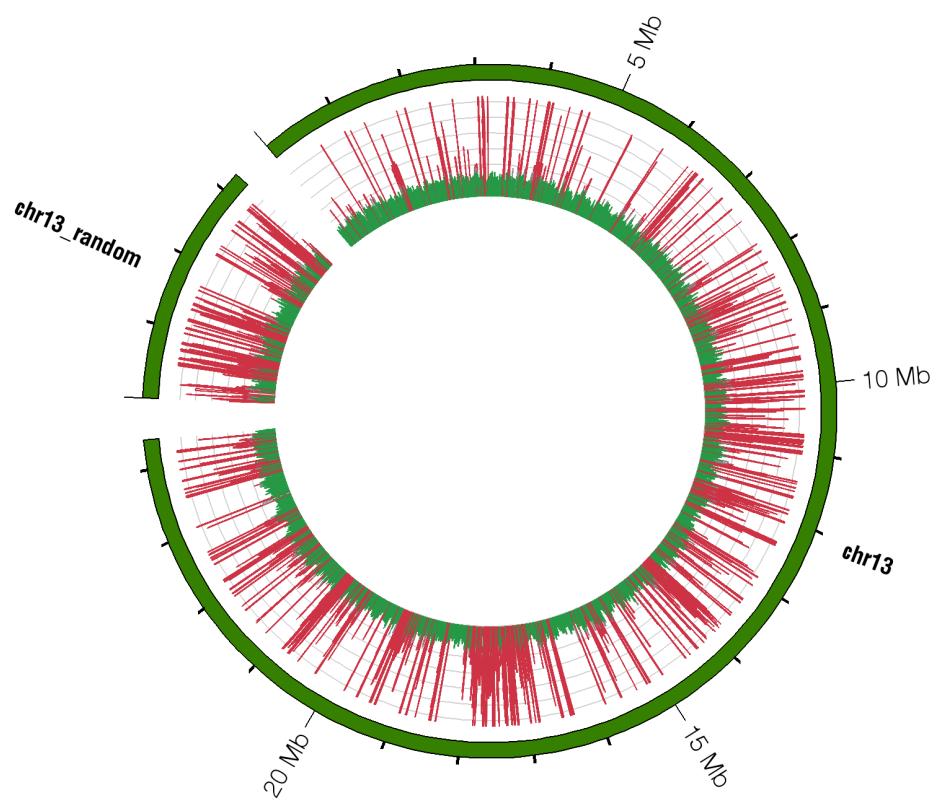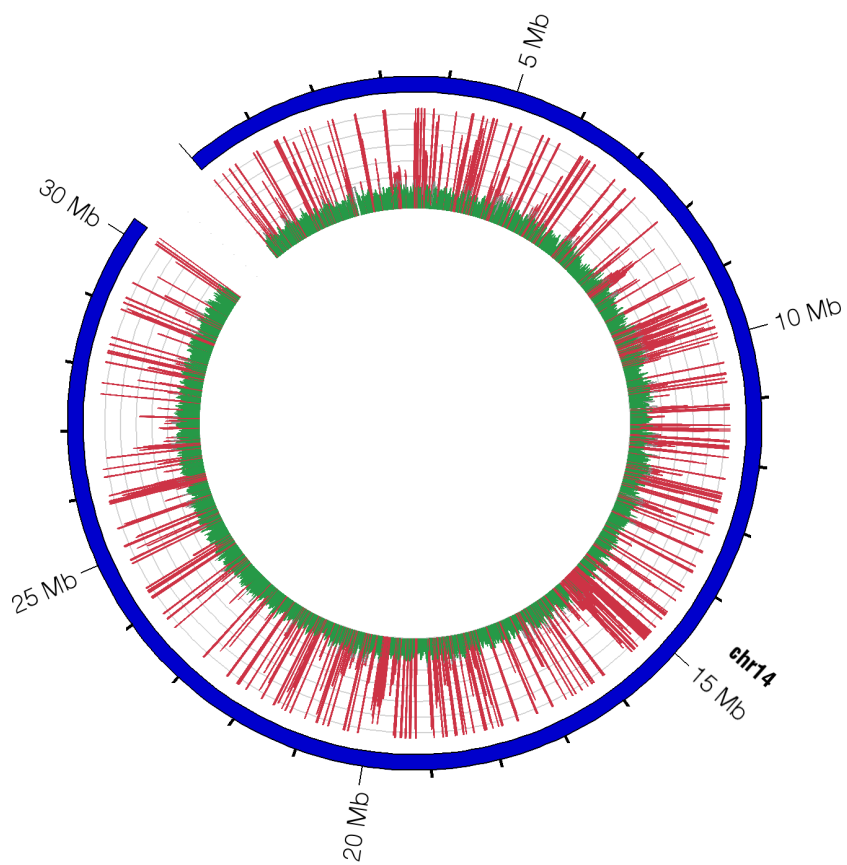

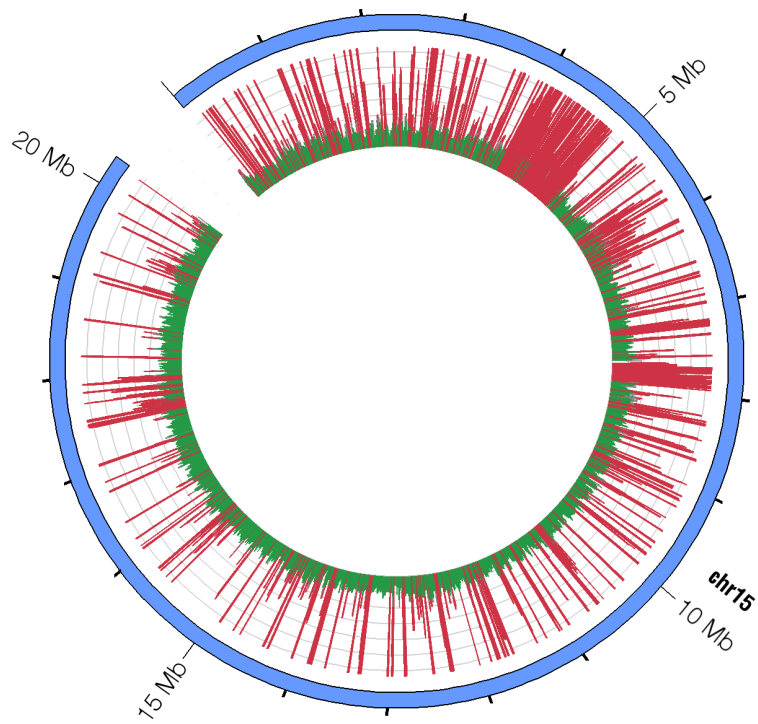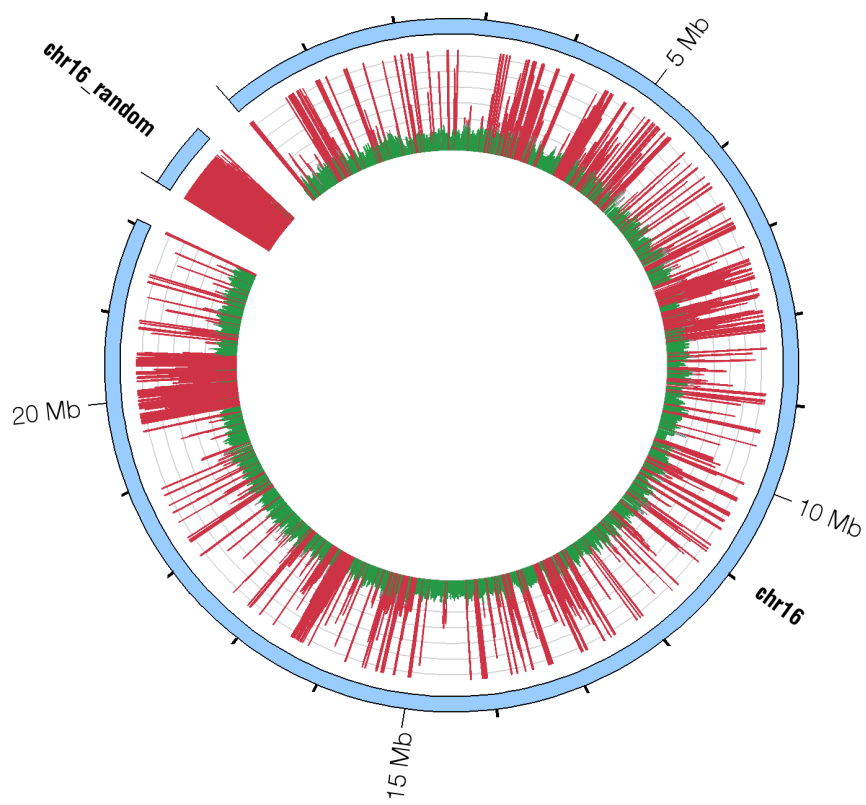

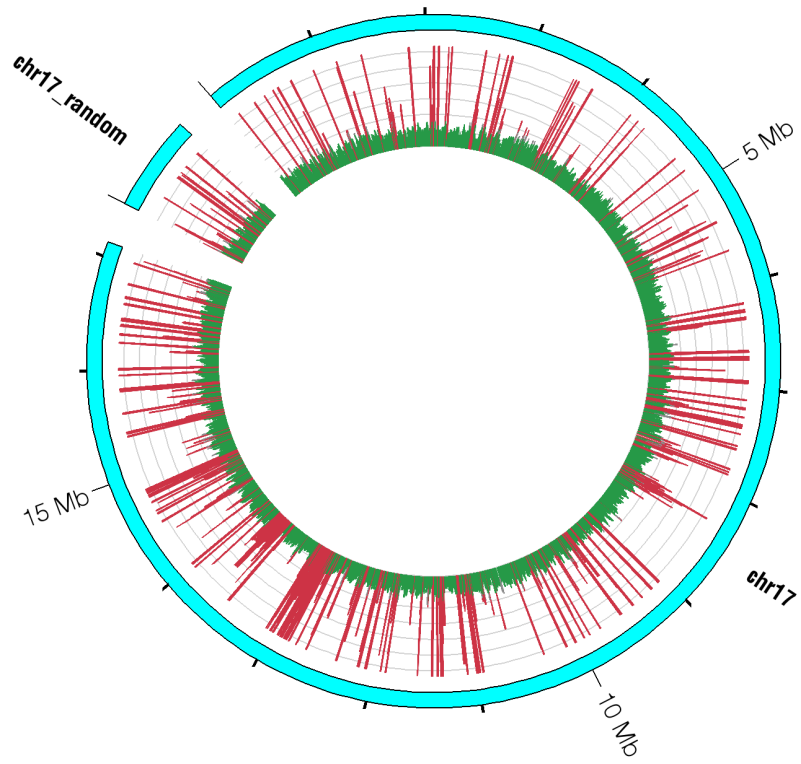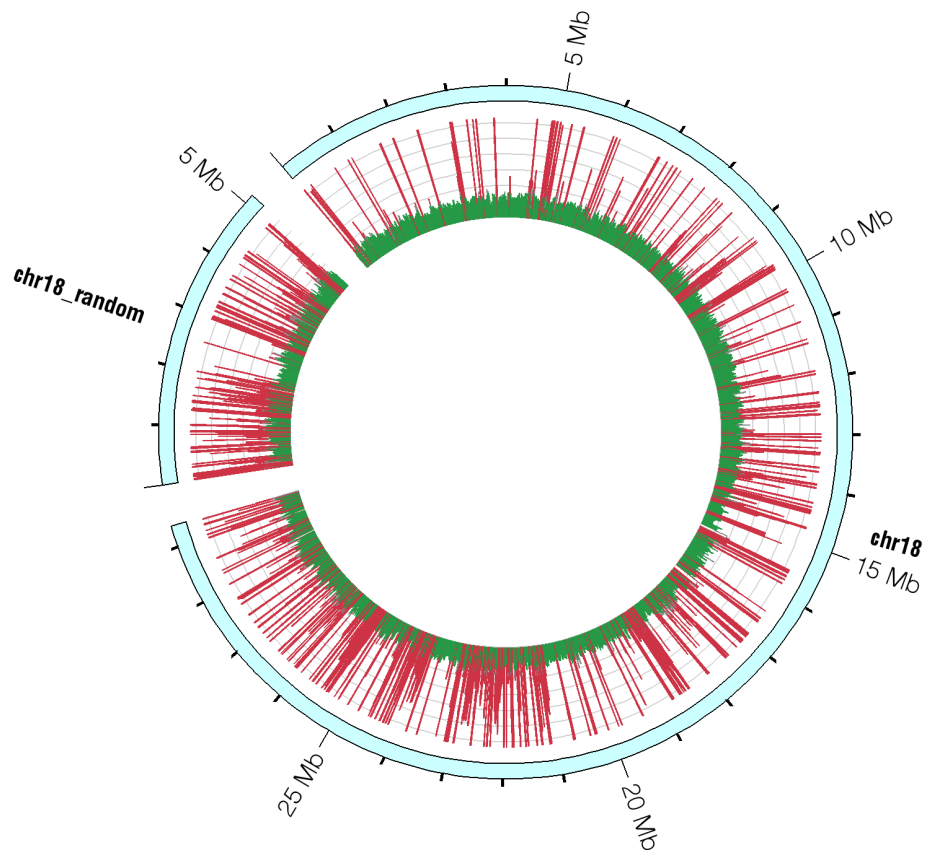

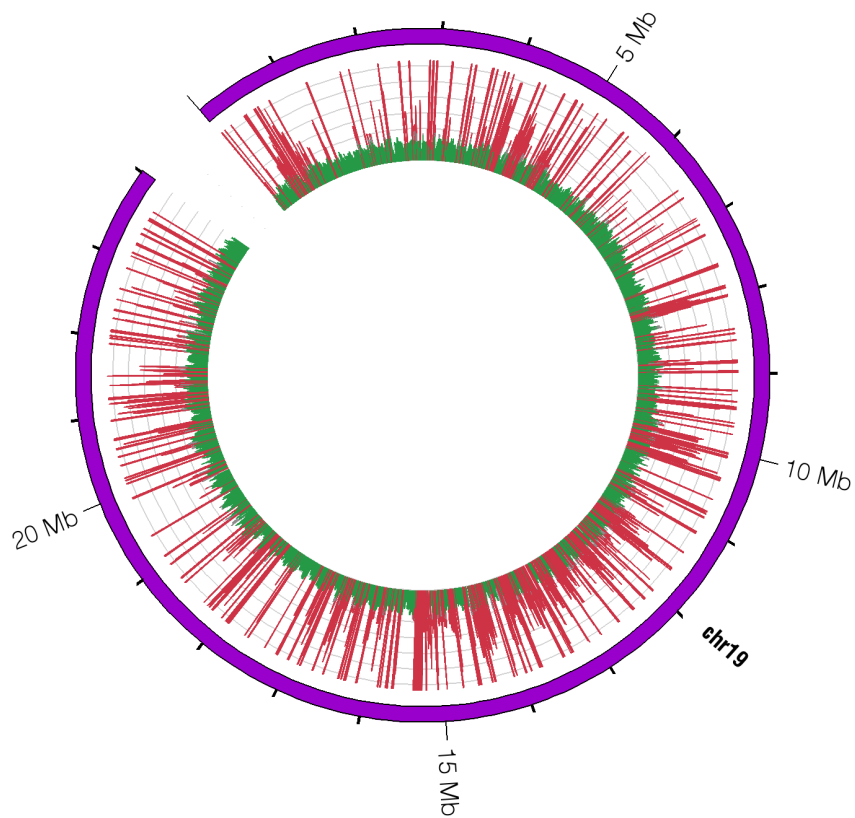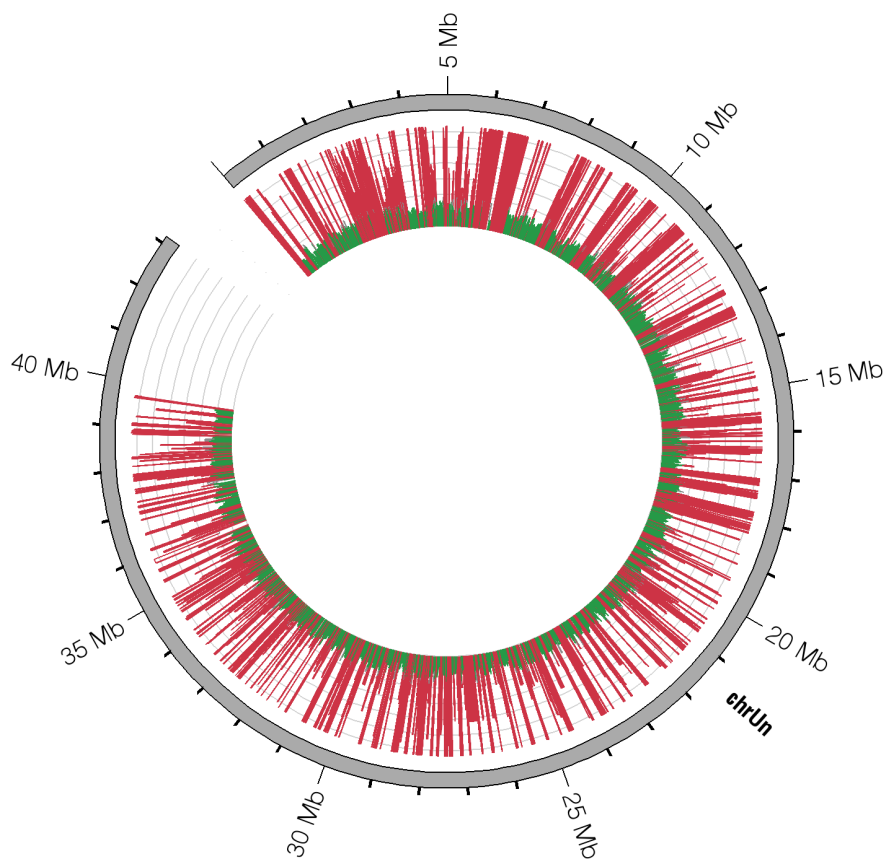

Supplement: Additional file 6 — WSSD coverage of Vitis vinifera chromosomes. The graphs illustrate the WSSD coverage of all Vitis vinifera chromosomes and were produced using the Circos tool. WSSD negative, borderline and positive windows are represented by green, gray and red colored bars, respectively. Last segment of chrUn sequence misses WSSD coverage values as it is composed of blocks, spaced out by gaps, too short to calculate the WSSD coverage on 5 kub windows. [file 1471-2164-12-436-S6.PDF]

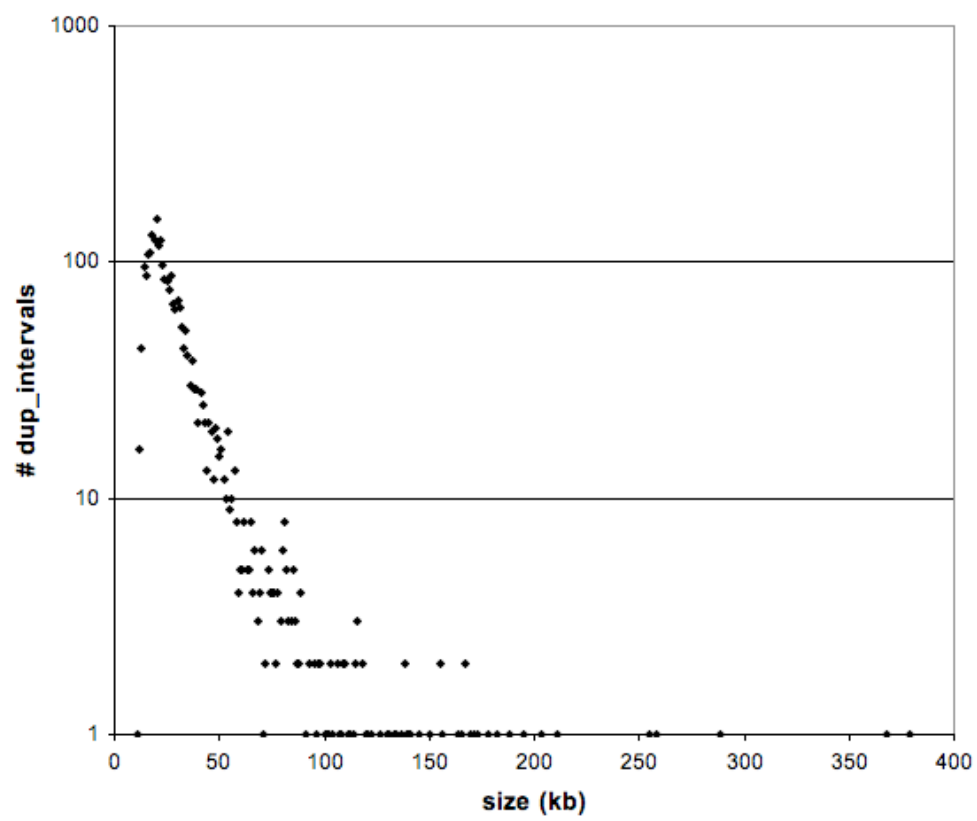

**Distribution size of duplicated intervals.**

Supplement: Additional file 8 — Distribution size of duplicated intervals. The graph shows the number of duplicated intervals according to their size. [file 1471-2164-12-436-S8.PDF]
