# Supplementary material for: Analysis of high-identity segmental duplications in the grapevine genome
Source: BMC Genomics. 2011 Aug 26;12:436. doi: 10.1186/1471-2164-12-436 (PMC3179966; doi:10.1186/1471-2164-12-436)
Supplement: Additional file 4 — Supplemental Note. The note reports the test of five different statistical models to identify the most appropriate one describing the WSSD coverage data. [file 1471-2164-12-436-S4.PDF]

Supplemental Note

Analysis of high-identity segmental duplications in the grapevine genome

Giuliana Giannuzzi, Pietro D'Addabbo, Marica Gasparro, Maurizio Martinelli, Francesco N Carelli, Donato Antonacci, Mario Ventura

We tested five statistical models to find the most appropriate to describe the read depth over the 5 kub (kilo of unmasked bases) windows (WSSD coverage) in the 39 single BAC-anchored loci. We replicated the models for three data sets obtained from different masking settings (div10_low, nodiv_low, and nodiv_N) (see Main Text for details). We used the RooFit tool of the software ROOT [1] to show the best fit (maximum likelihood) to the observed WSSD coverage data for each statistical model. The fit program evaluates the likelihood L comparing the probability density function (PDF) to the data set. The parameters of the best fit are retrieved by the minimization of the function -logL, performed by the MINUIT software [2]. In addition, for each tested distribution we calculated the reduced χ^2_{red}, which is defined as:

$$\chi^2_{red} = \chi^2 / \nu = 1/\nu \cdot \sum_{i=1}^{n} \left(O_i - N_i\right)^2 / \sigma_i^2$$

where ν is the number of degrees of freedom of the fit, O_i and σ_i are the number of events observed in the ith bin and its error, and N_i is the number of events predicted by the model in the ith bin. Large values of χ^2_{red} indicate a model poorly describing the observed data, while perfect agreement is found when $\chi^2_{red} = 1$; if $\chi^2_{red} < 1$, the model is over-fitting the data [3].

In regard to the Lander-Waterman theory reporting that sequencing coverage follows a Poisson distribution [4], we first fitted the three sets of WSSD coverage values with a Poisson distribution (**Figure SN1**). However, none of the data sets were distributed in a Poisson fashion. χ^2_{red} values were 565.4, 751.7 and 87.6 for the three masking

settings, respectively. This outcome reveals that whereas the Poisson distribution describes the genome coverage, which is the result of the DNA sequencing process, it is not appropriate for the WSSD coverage, which is the result of an analysis through sequence alignment.

We tested a normal distribution, having as mean and width the average and standard deviation, respectively, of the whole data sample (**Figure SN2**). The resulting curves did not properly describe any of the three data samples (χ^2_{red} values equal to 39.9, 35.6 and 36.0 for the three masking settings, respectively), since the high read depths of a few windows affected the average and standard deviation values. We then tried fitting the three data sets with a normal distribution using the RooFit tool, which gives the best Gaussian fitted to the data points (**Figure SN3**). Neither this model was adequate (χ^2_{red} values equal to 23.4, 22.4 and 21.4).

We suspected that the windows in the data samples with a high WSSD coverage value were due to the presence of duplicated segments too small to be detected by FISH and/or the presence of repetitive elements left unmasked. The frequencies showed peaks at coverage values equal to two, three and four times the data mode, which might correspond to regions present in two, three and four copies in the genome, respectively. To solve this multiplicity, we fitted a model made of four Poisson distributions (P_1, P_2, P_3 and P_4) to the WSSD coverage data sample for each masking setting (**Figure SN4**). P_2, P_3 and P_4 curves had a mean value 2, 3 and 4 times the P_1 mean value, respectively. This model arranges the mean value of the following Poisson distributions (P_2, P_3, and P_4) as related to the values of the first one (P_1): $\lambda_{Pn}=n\lambda_{P1}$. The PDF is defined as:

$$PDF = \sum_{n=1}^{4} f_n P_n(n\lambda), \text{ with } \sum_{n=1}^{4} f_n = 1$$

where λ is the mean value of Poisson distribution P_1 (n=1). The sum of areas under all Poisson curves is required to equal 1. The χ^2_{red} values were 11.8, 9.2 and 12.7 for the three masking settings, respectively. The P_1 distribution did not reproduce the observed data since they were more spread out around the mean. Therefore, this model was also not appropriate.

Finally, we fitted a model made of four Gaussians (G_1, G_2, G_3 and G_4) that suitably described the data points (**Figure SN5**). G_2, G_3 and G_4 curves had an average 2, 3 and 4 times the G_1 average (avg) and a standard deviation √2, √3 and √4 times the G_1 standard deviation (sd), respectively. The PDF used to describe this model is defined as:

$$PDF = \sum_{n=1}^{4} f_n G_n\left(n \cdot avg, \sqrt{n} \cdot sd\right), \text{ with } \sum_{n=1}^{4} f_n = 1$$

where avg and sd equal the average and standard deviation values of Gaussian G_1 (n=1). The sum of the areas under all Gaussian curves is required to equal 1. The χ^2_{red} values of this model were 5.2, 3.0 and 0.8 for the three masking settings, respectively.

Following the above tests and assessments, we considered the model made of the four Gaussian distributions as the most appropriate to describe the WSSD coverage values in the single-copy control regions, represented by the 39 single BAC-anchored loci. Gaussian G_1 described the values of single-copy windows. We then designated the windows with a WSSD coverage less than or equal to two sd above the avg (97.7% of windows in one copy) as “single”; the windows with a WSSD coverage greater than two sd above the avg and less than or equal to three sd above the avg (2.1% of windows in one copy) as “borderline”; and the windows with a WSSD coverage greater than three sd above the avg (0.1% of windows in one copy) as “duplicated”.

Notes for all SN Figures

The graphs show the fit results for the tested statistical models of the WSSD coverage data samples from 39 single BAC-anchored loci obtained using the "div10_low", "nodiv_low" and "nodiv_N" masking settings. The observed WSSD coverage data are shown as black dots. The four Poisson (P_1, P_2, P_3 and P_4) and Gaussian (G_1, G_2, G_3 and G_4) distributions are drawn in green, red, yellow and magenta, respectively. The final fit (single Poisson and single Gaussian distribution, and the sum of four Poisson and four Gaussian distributions) is drawn in blue. Values of the distribution parameters are shown in the box. χ^2_{red} values are displayed. On top, the normalized residuals distribution (Pull=$(N_{data}-N_{fit})/\sigma_{data}$, N and σ being the number of events and the error for each bin) is shown. When the pull distribution is fully between -3 and 3, fluctuations are only statistical.

div10_low

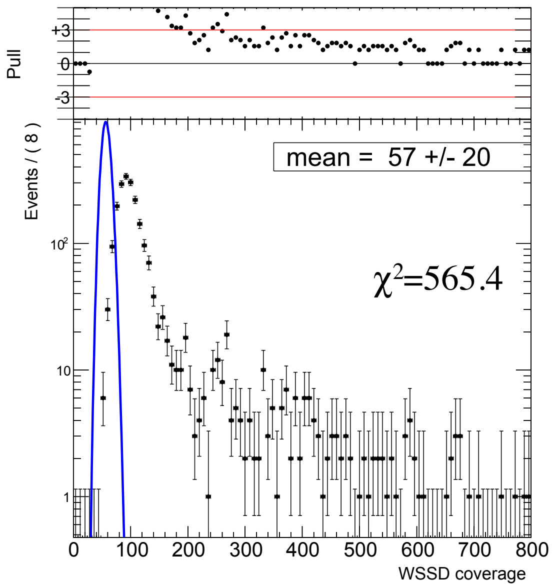

nodiv_low

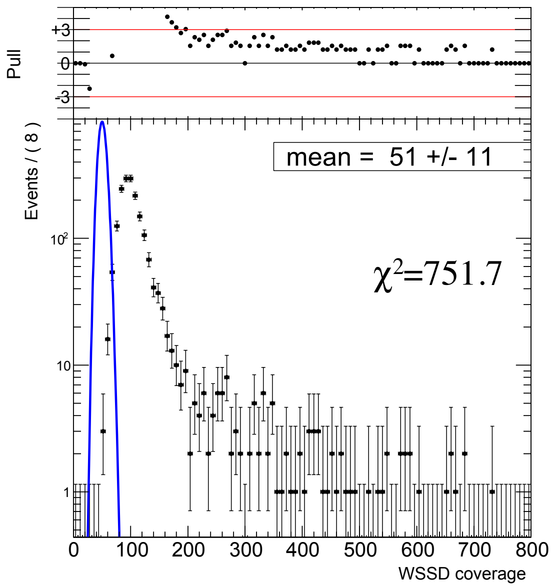

nodiv_N

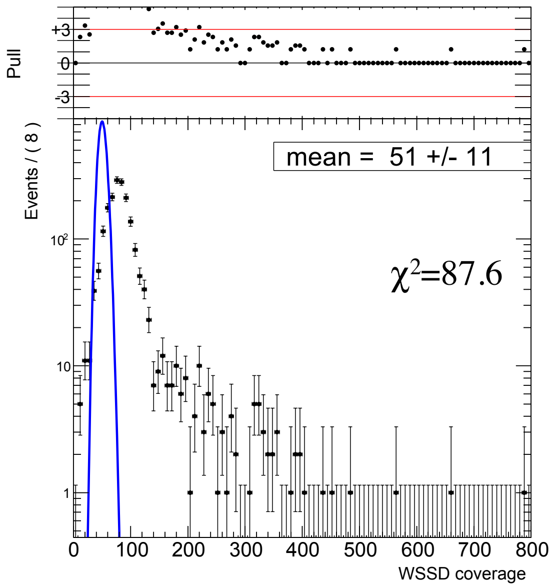

Figure SN1. Fit results of WSSD coverages to a Poisson distribution.

div10_low

nodiv_low

nodiv_N

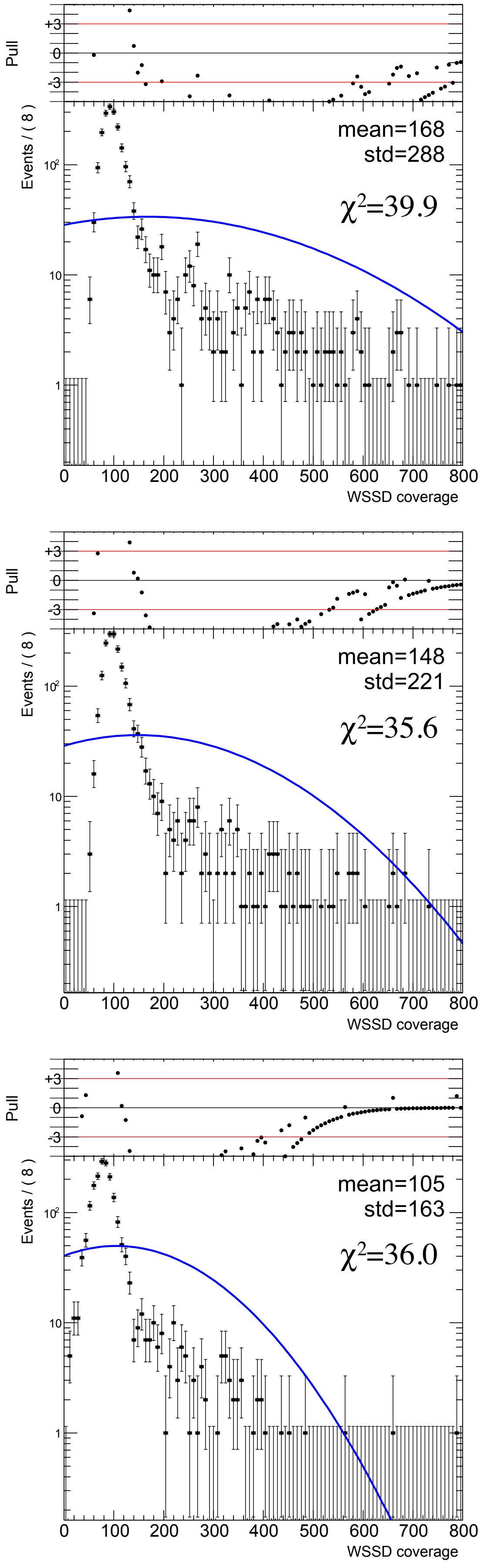

Figure SN2. Fit results of WSSD coverages to a Gaussian distribution with mean and width equal to the average and standard deviation of the data sample.

div10_low

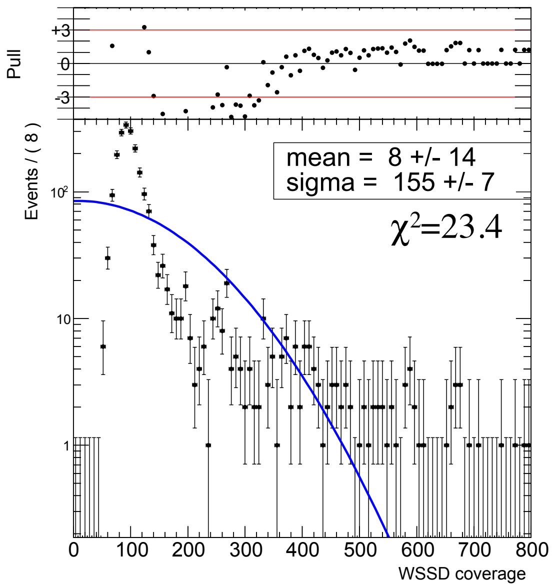

nodiv_low

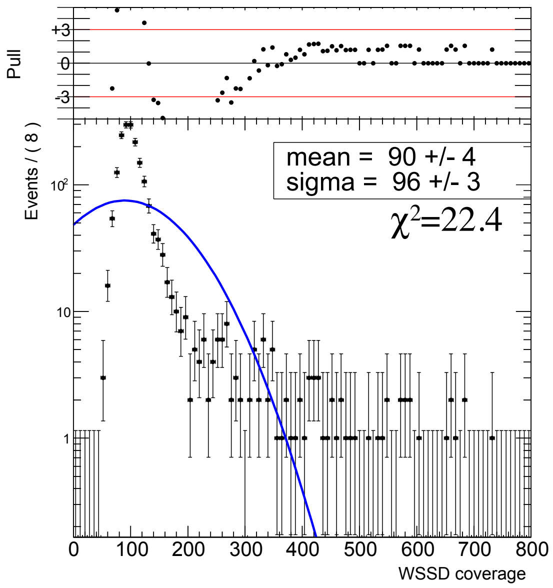

nodiv_N

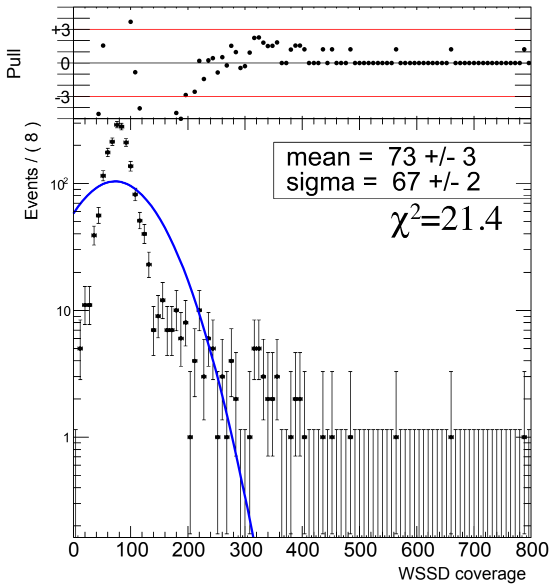

Figure SN3. Fit results of WSSD coverages to a Gaussian distribution.

div10_low

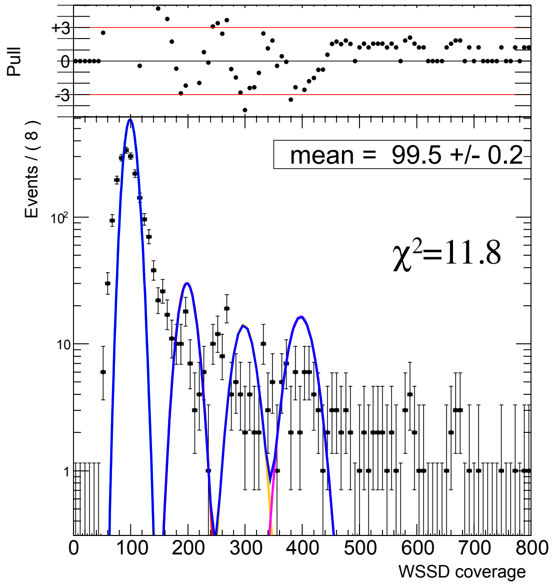

nodiv_low

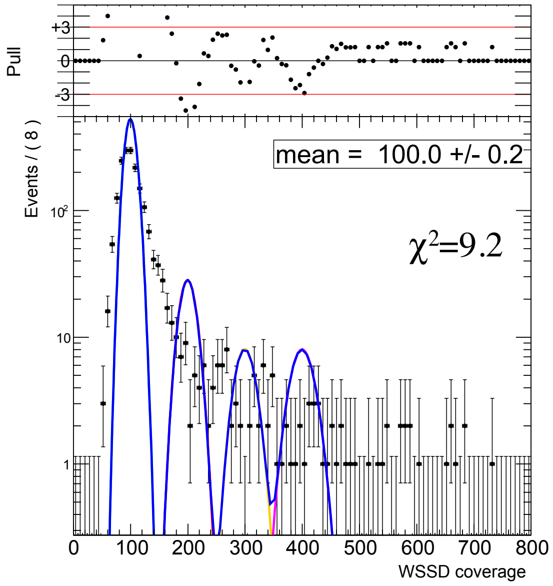

nodiv_N

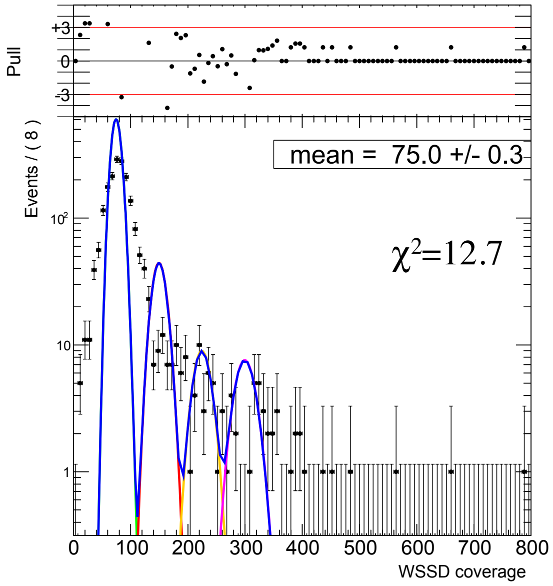

Figure SN4. Fit results of WSSD coverages to a model of four Poisson distributions.

div10_low

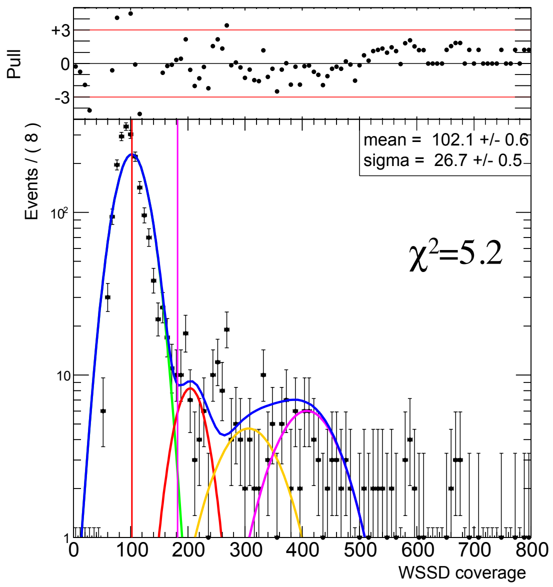

nodiv_low

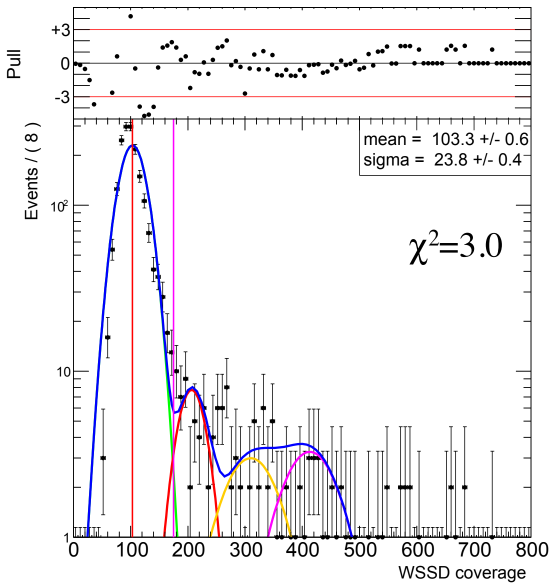

nodiv_N

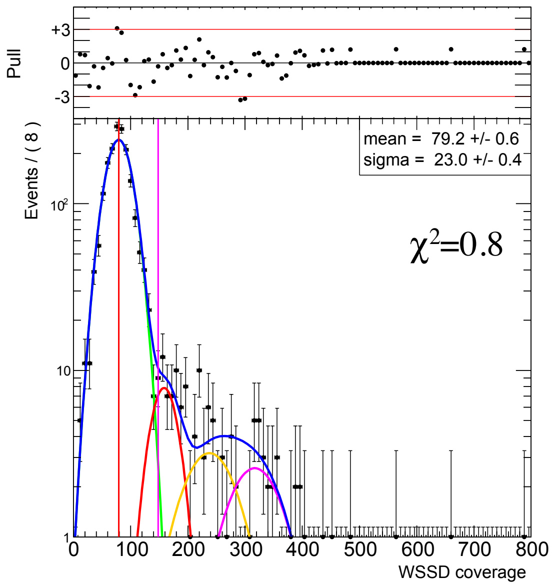

Figure SN5. Fit results of WSSD coverages to a model of four Gaussian distributions.

Supplemental References

1. Antcheva I, Ballintijn M, Bellenot B, Biskup M, Brun R, Buncic N, Canal P, Casadei D, Couet O, Fine V, et al: **ROOT — A C++ framework for petabyte data storage, statistical analysis and visualization.** *Comput Phys Commun* 2009, **180:**2499-2512.
2. James F, Roos M: **MINUIT - a system for function minimization and analysis of the parameter errors and correlations.** *Comput Phys Commun* 1975, **10:**343-367.
3. Cowan G: *Statistical Data Analysis.* Clarendon Press, Oxford; 1998.
4. Lander ES, Waterman MS: **Genomic mapping by fingerprinting random clones: a mathematical analysis.** *Genomics* 1988, **2:**231-239.
